# Use of an Activated Beta-Catenin to Identify Wnt Pathway Target Genes in *Caenorhabditis elegans*, Including a Subset of Collagen Genes Expressed in Late Larval Development

**DOI:** 10.1534/g3.113.009522

**Published:** 2014-02-25

**Authors:** Belinda M. Jackson, Patricia Abete-Luzi, Michael W. Krause, David M. Eisenmann

**Affiliations:** * Department of Biological Sciences, University of Maryland Baltimore County, Baltimore, Maryland 21250; † Laboratory of Molecular Biology, National Institute of Diabetes and Digestive and Kidney Diseases, National Institutes of Health, Bethesda, Maryland 20892-0510

**Keywords:** Wnt, *C. elegans*, collagen, gene expression, cell signaling

## Abstract

The Wnt signaling pathway plays a fundamental role during metazoan development, where it regulates diverse processes, including cell fate specification, cell migration, and stem cell renewal. Activation of the beta-catenin−dependent/canonical Wnt pathway up-regulates expression of Wnt target genes to mediate a cellular response. In the nematode *Caenorhabditis elegans*, a canonical Wnt signaling pathway regulates several processes during larval development; however, few target genes of this pathway have been identified. To address this deficit, we used a novel approach of conditionally activated Wnt signaling during a defined stage of larval life by overexpressing an activated beta-catenin protein, then used microarray analysis to identify genes showing altered expression compared with control animals. We identified 166 differentially expressed genes, of which 104 were up-regulated. A subset of the up-regulated genes was shown to have altered expression in mutants with decreased or increased Wnt signaling; we consider these genes to be *bona fide C. elegans* Wnt pathway targets. Among these was a group of six genes, including the cuticular collagen genes, *bli-1col-38*, *col-49*, and *col-71*. These genes show a peak of expression in the mid L4 stage during normal development, suggesting a role in adult cuticle formation. Consistent with this finding, reduction of function for several of the genes causes phenotypes suggestive of defects in cuticle function or integrity. Therefore, this work has identified a large number of putative Wnt pathway target genes during larval life, including a small subset of Wnt-regulated collagen genes that may function in synthesis of the adult cuticle.

The Wnt signaling pathway is one of the most widely used and essential extracellular signaling mechanisms in metazoans, and components of this pathway are conserved from hydra to humans. Wnts are secreted ligands that regulate a vast number of developmental processes in vertebrates and invertebrates, including cell migration, cell polarity, fate specification, axon guidance, synaptogenesis, and stem cell differentiation and renewal (Clevers 2006; Cadigan and Peifer 2009; MacDonald *et al.* 2009; Clevers and Nusse 2012). Wnts mediate these effects by binding to a conserved coreceptor complex and activating one of several downstream signaling cascades. The most well-studied of these pathways is dependent on the activity of the transcription factor beta-catenin, and is known as the Wnt beta-catenin−dependent signaling pathway or the “canonical” Wnt pathway.

The Wnt beta-catenin−dependent pathway appears to function substantially similarly across the metazoan clade but has been most well characterized in Drosophila and vertebrate species (for review, see Clevers 2006; Cadigan and Peifer 2009; MacDonald *et al.* 2009; Clevers and Nusse 2012). In this pathway, lipid-modified Wnt ligands are secreted from the sending cell and are bound on the surface of a receiving cell by a coreceptor consisting of a member of the seven transmembrane Frizzled receptor family and a single transmembrane receptor of the low-density lipoprotein related protein 5/6 class. Ligand binding regulates the formation of the downstream effector of this pathway, a heterodimeric transcription factor consisting of a member of the TCF/LEF (T-cell factor/lymphoid enhancer factor) family of HMG box-containing DNA binding proteins, and the transcription activator beta-catenin. In the absence of signal, the TCF family member is bound at the promoters of Wnt-regulated genes, where it interacts with transcriptional repressors to keep target gene expression off or low. Meanwhile, beta-catenin is bound by a “destruction complex” consisting of the scaffold proteins Axin and APC and the kinases GSK3beta and CK1, which phosphorylate beta-catenin on conserved sites in its amino terminus, marking it for ubiquitin-mediated degradation by the proteasome. When Wnt ligands are bound, the destruction complex is disrupted by recruitment of some it its members to the activated LRP receptor, which allows beta-catenin to escape phosphorylation and degradation. The stabilized beta-catenin protein translocates into the nucleus and interacts with TCF/LEF, displacing the transcription repressors and recruiting the transcription machinery to activate expression of Wnt-regulated target genes.

The physiological and developmental effects of Wnt signaling on receiving cells is attributable to specific target gene expression, and Wnt pathway targets have been identified in vertebrates and invertebrates by genetic, molecular, bioinformatic, and genomic approaches (Vlad *et al.* 2008; Nusse 2013). Target genes encode a variety of proteins, including downstream terminal effectors such as proteins controlling the cell cycle or cell migration, transcription factors that presumably activate additional indirect target genes, and secreted molecules that may mediate further nonautonomous signaling events. Most target genes contain one or more binding sites for TCF/LEF proteins, which prefer a common core DNA binding site with the consensus CTTTGWW, although other divergent binding sites have also been characterized (Cadigan 2012).

We have previously examined the role of Wnt signaling during larval development in the nematode *C. elegans*. Beta-catenin dependent Wnt signaling is more complex in *C. elegans*, as this species has two beta-catenin dependent Wnt signaling pathways (Eisenmann 2005; Jackson and Eisenmann 2012). A Wnt/beta-catenin dependent “canonical” (WBC) pathway similar to that described in other species is present and uses the beta-catenin BAR-1 and the single worm TCF family member, POP-1. The WBC pathway regulates several developmental events during worm larval life, including regulation of cell fate specification by the vulval precursor cells (VPCs), the posterior hypodermal cell P12, and the progeny of the Q neuroblasts (Eisenmann 2005; Jackson and Eisenmann 2012). In all three of these processes, Wnt signaling appears to regulate expression of one of the *C. elegans* Hox cluster genes.

*C. elegans* also uses a second beta-catenin dependent pathway known as the Wnt/beta-catenin asymmetry (WBA) pathway, which is a major regulator of cell fate specification between daughters of asymmetric cell divisions during development (Mizumoto and Sawa 2007; Jackson and Eisenmann 2012). The WBA pathway uses POP-1 with two different beta-catenin proteins, SYS-1 and WRM-1. Here, Wnt binding leads to the activation of both a Wnt signaling pathway and a mitogen-activated protein kinase/Nemo-like kinase pathway, which converge to cause both a WRM-1−dependent decrease in POP-1 level and an increase in SYS-1 level in the nucleus of only one daughter cell of an asymmetric division. In this cell, POP-1 and SYS-1 heterodimerize and activate target gene expression, whereas in the other daughter cell POP-1 is bound to transcription repressors, so target genes are not activated. The WBA pathway regulates many events in embryonic and larval development that involve differences in fate between daughters of an asymmetric division, including specification of the progeny of the EMS blastomere at the four cell stage, determination of the somatic gonad progenitor cells, and the stem cell−like division of the lateral hypodermal seam cells during larval growth (Mizumoto and Sawa 2007; Jackson and Eisenmann 2012).

Although many details of these two *C. elegans* Wnt pathways are known, our knowledge of target genes regulated by these pathways is minimal. Several *bona fide* targets of the WBA pathway have been identified by molecular genetic methods: *ceh-10*, *ceh-22*, *egl-18*, *elt-6*, *end-1*, *end-3*, *ceh-22*, *ceh-10*, and *psa-3* (Streit *et al.* 2002; Maduro *et al.* 2005; Shetty *et al.* 2005; Arata *et al.* 2006; Lam *et al.* 2006; Bertrand and Hobert 2009; Gorrepati *et al.* 2013). Known targets of the WBC pathway are three *C. elegans* Hox genes (*mab-5*, *egl-5*, *lin-39)*; however, it has not been shown that regulation is direct; in fact, no direct targets of the WBC pathway have been identified to date (Eisenmann *et al.* 1998; Jiang and Sternberg 1998; Maloof *et al.* 1999). Interestingly, all of the Wnt target genes identified to date by genetic methods encode transcription factors, suggesting they may not be the terminal effectors of signaling in these cells. Analysis of the POP-1 binding sites in WBA target genes shows that the *C. elegans* TCF protein binds to the consensus CTTTGWW site, but unlike TCFs in other species, POP-1 also binds DNA sites with a T in the first position (Jackson and Eisenmann 2012). Given the GC content of the *C. elegans* genome (*C. elegans* Sequencing Consortium 1998), the modified POP-1 consensus site (YTTTGWW) is predicted to be present randomly at least once per kilobase in the genome, precluding easy bioinformatics identification of POP-1 target sites.

To begin to address the deficit in our knowledge of the targets of the WBC pathway, we undertook a microarray analysis to identify genes that showed altered expression upon conditional activation of Wnt signaling in living animals during a defined stage of larval life. By comparing gene expression in animals expressing a dominant, activated beta-catenin protein to that in control animals, we identified 166 differentially expressed genes, of which 104 were up-regulated and 62 were down-regulated. Because Wnt signaling usually acts to up-regulate target gene expression, we consider these 104 genes to be candidate Wnt pathway targets in the *C. elegans* hermaphrodite larva. Unlike the genes identified by molecular genetic approaches, only one of these genes encodes a known transcription factor, suggesting they may be downstream effectors of Wnt signaling in the worm. A subset of the up-regulated genes were validated by quantitative polymerase chain reaction (qPCR) analysis, showing altered expression in strains with conditional activation or inhibition of Wnt signaling and in Wnt pathway mutants with decreased or increased Wnt signaling. Among these was a group of six genes, including the cuticular collagen encoding genes, *bli-1col-38*, *col-49*, and *col-71*. We found that these genes show a peak of expression in the mid L4 stage during normal development, and expression at this time is altered in Wnt pathway mutants. The L4 expression of these genes suggests they may be expressed for use in the adult cuticle, and, consistent with this, reduction of function for several of the genes leads to phenotypes suggestive of defects in cuticle function or integrity. Therefore, this work has identified a large number of putative Wnt pathway target genes during larval life, including a small subset of Wnt-regulated collagen genes that may function in synthesis of the adult cuticle.

## Materials and Methods

### *C. elegans* strains

Methods for *C. elegans* culture, maintenance, and genetic manipulation are described in Brenner (1974). References for genes and alleles used in this work are Riddle *et al.* (1997) and Wormbase (www.wormbase.org) (Harris *et al.* 2010; Yook *et al.* 2012). Wild-type refers to *C. elegans* Bristol variety (N2). Mutant alleles used in this work include the following: (LG I): *unc-29(e193)*, *daf-16(mgDf50)*, *pry-1(mu38)*; (LG II): *sptf-2(tm1130)*, *bli-1(e769)*, *F08G2.7(ok1161)*; (LG III): *tag-164(o)*, *unc-119(ed3)*; (LG IV): *dpy-20(e1282)*; (LG V): *him-5(e1490)*, and (LG X): *bar-1(ga80)*. Strains were maintained at 20° except where otherwise noted.

Three heat-shock inducible strains were used in this work: (1) the *hs*::*ΔNTbar-1* strain (KN53; gift of Rik Korswagen) contains the array *huIs7* [pHCK64 *(hsp16.2::MycΔNTbar-1)*; *mec-7*::*GFP*; *dpy-20(+)*], which expresses a Myc-tagged truncated BAR-1 protein expressed from heat shock promoter (Gleason and Eisenmann 2010); (2) the *hs*::*control* strain carries the array *deIs14*, which contains the empty heat shock vector pPD49.78; and (3) the *hs*::*ΔNTpop-1* strain carries the array *deIs15*, which contains pHCK28 (gift of Rik Korswagen), which expresses a truncated POP-1 protein expressed from the heat shock promoter in pPD49.78 [the same plasmid was used previously to create array *huIs4* (Korswagen *et al.* 2000)]. The latter two strains were generated via microinjection into mutant strain *dpy-20(e1282*) of the heat shock construct (50 ng/μL), *mec-7*::*GFP* (Ch’ng *et al.* 2003) (50 ng/μL), and *dpy-20(+)* plasmid pMH86 (Han and Sternberg 1991) (100 ng/μL). Strains carrying YFP translational reporters *col-38(p)*::*YFP*, *col-49(p)*::*YFP*, and *bli-1(p)*::*YFP* were made by injecting reporter plasmid DNA (100 ng/μL), which also contains the *unc-119(+)* gene (Maduro and Pilgrim 1995) into *unc-119(ed3)*. Array integration was performed by gamma irradiation and screening of progeny for >95% *GFP* expression penetrance; in each case, at least three separate lines were backcrossed and characterized.

### Heat shock protocol

Embryos from *hs*::*ΔNTbar-1*, *hs*::*ΔNTpop-1*, and *hs*::*control* strains were collected by hypochlorite treatment and hatched in H_2_O for 20 hr. Arrested L1 animals were transferred to nematode growth media plates with OP50 *E. coli* and allowed to develop to the L2/L3 molt (24 hr). Plates were transferred to 38° for 30 min, then returned to 20°. Worms used for microarray analysis, qPCR validation, and DAF-16 dependence were collected after 1-hr recovery; worms used for YFP reporter analysis were collected at the L3, L4, and adult stages as indicated. An aliquot of *hs*::*ΔNTbar-1* heat-shocked animals was allowed to develop to the adult stage and showed >90% Multivulva (Muv) phenotype, an indication of WBC pathway activation (Gleason *et al.* 2002).

### RNA processing and Affymetrix microarray analysis

Total RNA for microarray analysis was isolated from a minimum of three biological replicates using a QIAGEN RNAeasy mini kit following dissociation with 2.4-mm zirconia beads (BioSpec Products) and either a Savant BIO-101/FP120 homogenizer or a Miltenyi gentleMACS Dissociator. cDNA was generated using the Bio-Rad iScript cDNA synthesis kit. For Affymetrix microarray hybridization, 5 μg of total RNA from three biological replicates of heat-shock treated strains was synthesized into separate pools of double-stranded cDNA. cDNA was *in vitro* transcribed, fragmented, and labeled to produce biotinylated cRNA hybridization targets, which were hybridized to oligonucleotide tags on the Affymetrix *C. elegans* Genome Array Genechip. Data from this article have been deposited with the NCBI Gene Expression Omnibus in dataset GSE51502.

Signal detection and statistical analysis were performed using the Affymetrix MAS 5 software for detection calls and the Partek Genomics Suite software for RMA and statistical analysis. Comparisons were made between the averaged signal amounts of three *hs*::*control* data sets and each of the three experimental *hs*::*ΔNTbar-1* data sets. Criteria used for selection of BAR-1 responsive gene targets were (1) at least a twofold change in signal response to ΔNTBAR-1 overexpression compared with control average, (2) concurrence in directional change for at least two of the three ΔNTBAR-1 induced replicates (*e.g*., increase or decrease of signal), and (3) an analysis of variance p-value of ≤ 0.05. GO term analysis of the target gene list was carried out using the Database for Annotation, Visualization, and Integrated Discovery (DAVID; david.abcc.ncifcrf.gov) (Huang *et al.* 2009a,b) and FuncAssociate at http://llama.mshri.on.ca/cgi/func1/funcassociate (Berriz *et al.* 2009).

### qPCR analysis

For 100 putative Wnt up-regulated genes identified by microarray analysis, qPCR validation was performed on a minimum of two biological replicates; genes that showed preliminary validation (including all 22 class I and II genes) were subject to additional analysis of independent biological replicates for a minimum of three per gene. qPCR analysis was performed on a Bio-Rad iCycler Multicolor System using three technical replicates for all reactions. Relative expression ratios were calculated via the Δ,Δ^Ct^ equation (Pfaffl 2001) using either *gpd-2* or *ama-1* as reference gene. For validation of BAR-1 responsive genes, comparisons were made between synchronized *hs*::*control*, *hs*::*ΔNTbar-1*, and *hs*::*ΔNTpop-1* animals subjected to a heat shock at the L2/L3 molt and recovered for 60 min. For DAF-16 dependence, comparisons were made between synchronized *hs*::*ΔNTbar-1* and *hs*::*ΔNTbar-1;daf-16(mgDf50)* animals subjected to a heat shock at the L2/L3 molt and recovered for 60 min. For temporal expression analysis, arrested wild-type L1 animals were fed and allowed to develop at 25° over a 48-hr time span, with samples collected at 2-hr intervals. Ct (cycle threshold) values were determined for each sample and are presented in normalized units relative to the point of least expression, which was given an arbitrary value of one. For expression analysis in Wnt pathway mutants, comparisons were made between N2 animals and either *pry-1(mu38)* or *bar-1(ga80)* synchronized animals grown at 15° to the developmental time point corresponding to peak target gene expression (L1 larvae: *T26E4.4*, *srw-93*, *oac-30*; L2 larvae: *srw-93*, *oac-30*; L3 larvae: *oac-30*; L4 larvae: *col-38*, *col-49*, *col-71*, *bli-1*, *dao-4*, *dod-23*, *Y41C4A.11*, *T26E4.4*, *oac-30*, *tag-164(ok771)*; *y*oung Adults: *col-38*, *col-49*, *col-71*, *bli-1*, *dao-4*, *dod-23*, *Y41C4A.11*, *sptf-2*, *T26E4.4*, *oac-30*, *srw-93*, *tag-164(ok771)*).

### Construction and analysis of YFP transcriptional reporters

YFP transcriptional reporters were constructed using Invitrogen Gateway cloning technology (*col-38*, *col-49*, *col-71*, *bli-1*, *dao-4*, *Y41C4A.11*, *tag-164(ok771)*, and *Y43C5A.3)* or PCR fusion (*pry-1)*. For Gateway cloning, the 5′ intergenic region of the gene of interest, consisting of the sequence between the ATG translational start site and the next upstream gene, was PCR amplified and inserted upstream of two nuclear localization signals and the YFP coding sequence in pBJ101, an altered destination vector pDEST-YFP containing an *unc-119(+)* minigene. The intergenic region alone was insufficient to drive YFP expression in the *col-71* and *Y41C4A.11* reporters; therefore, we also included the first two and three introns of these genes, respectively. For fusion PCR, the 5′ intergenic region of *pry-1* was amplified from a genomic DNA template, and *2xNLS*::*YFP* was amplified from pPD107.94 (gift of A. Fire, Stanford University School of Medicine, Stanford, CA), to create *pry-1(p)*::*YFP*, which was coinjected with the *unc-119(+)*.

To examine the response of these reporters to ectopic Wnt signaling, integrated *col-38(p)*::*YFP*, *col-49(p)*::*YFP*, or *bli-1(p)*::*YFP* arrays were crossed into *hs-control*, *hs*::*ΔNTbar-1*, and *hs*::*ΔNTpop-1* backgrounds to create nine strains (both arrays were homozygous), which were subjected to the standard L2/L3 molt heat shock protocol and examined for YFP expression at various times using a Zeiss Axioplan 2 microscope, a Nikon DMX 1200 digital camera and Nikon ACT-1 software.

### RNA interference (RNAi) analysis

RNAi plasmids for *col-38*, *col-49*, *col-71*, *dao-4*, *sptf-2*, *tag-164(ok771)*, *pry-1*, *pop-1*, *F08G2.7*, and *Y41C4A.1* were obtained from Ahringer library (Kamath *et al.* 2001). The *bli-1* RNAi plasmid was generated by PCR amplification of a 200-bp fragment from N2 genomic DNA, which was ligated into feeding vector pPD129.36. Induction of dsRNA in *E. coli* strain HT115 (Timmons *et al.* 2001) was as described (Gleason *et al.* 2002). Synchronized L1 larvae grown on dsRNA producing bacteria were grown to the L3, L4, and young adult stages at 15°, 20°, and 25° and examined for obvious phenotypes.

### Cuticle integrity assays

The cuticle integrity of wild-type, mutant (*bar-1*, *bli-1*, *sptf-2*, *tag-164(ok771)*, and *F08G2.7)*, RNAi-treated (*col-38*, *col-49*, *col-71*, and *dao-4*) and RNAi-control animals was determined by permeability to Hoechst stain (Moribe *et al.* 2004). Animals were grown to adulthood on NGM plates with *E. coli* strain OP50, or on RNAi plates with HT115 bacteria expressing dsRNA for the appropriate gene or vector alone. Three-day-old adults were picked and incubated with gentle agitation in 10 μg/mL Hoechst 33258 (Sigma-Aldrich) for 15 min at room temperature, washed in M9 buffer, and analyzed for nuclear staining on a Zeiss Axioplan 2 microscope.

### Western blot analysis

Synchronized *hs*::*ΔNTbar-1* L1 larvae were grown to the L2/L3 molt (24 hr), given a heat shock (38°, 30 min), then returned to 20°. Samples were collected immediately following heat-shock and every hour for 8 hr and stored in lysis buffer at −80° until needed. Western blotting was by standard methods. MYC-tagged ΔNTBAR-1 was detected using HRP conjugated anti-c-MYC antibody (#A 5598; Sigma-Aldrich); actin loading control was detected using anti-β-Actin (# A 3854; Sigma-Aldrich).

### Primer sequences

The sequences of oligonucleotides used for plasmid synthesis and qPCR analysis are available in Supporting Information, Table S8.

## Results

### Use of a heat shock-expressed dominant beta-catenin to identify *C. elegans* genes responsive to Wnt pathway activation

**Figure 1:**
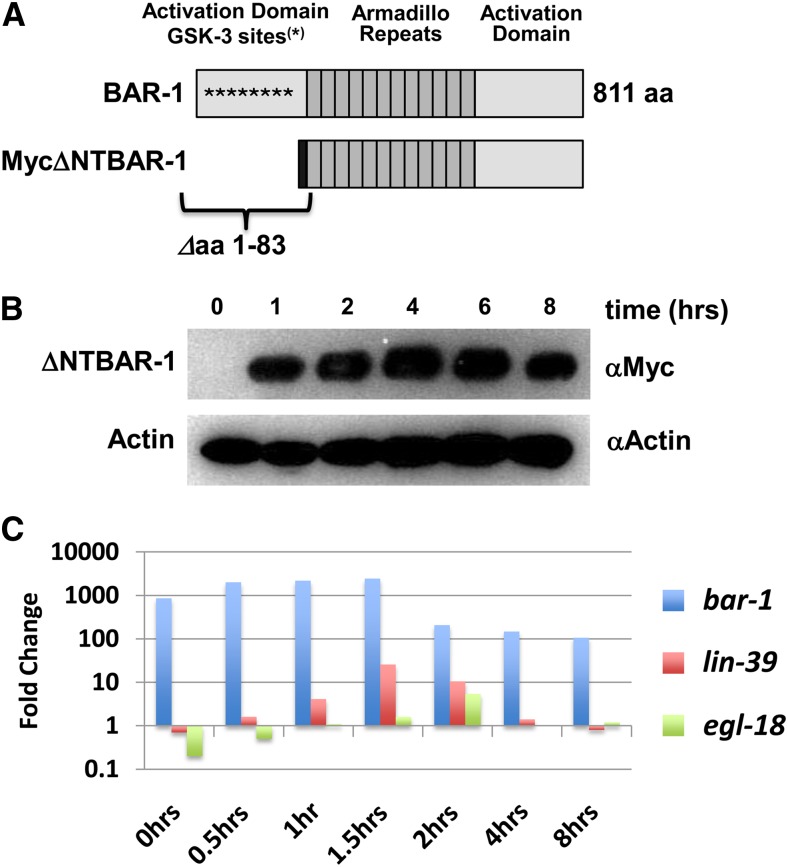
Expression of a truncated version of beta-catenin BAR-1 from the heat shock promoter increases target gene expression. (A) Diagram of the 811 amino acid wild-type BAR-1/beta-catenin protein (top), showing location of major motifs, including eight GSK-3 phosphorylation sites that are deleted in the ΔNTBAR-1 protein (bottom); this protein also incorporates a Myc epitope tag (dark box) at the amino terminus. (B) Western blot analysis showing expression of ΔNTBAR-1 protein in *hs*::*ΔNTbar-1* animals at indicated times after a 30-min heat shock. BAR-1 was detected using anti-Myc antibody. (C) qPCR analysis on transcripts for *bar-1*, *lin-39* [putative WBC pathway target (Eisenmann *et al.* 1998; Wagmaister *et al.* 2006)] and *egl-18* [LIN-39 target in VPCs (Koh *et al.* 2002)], at indicated times after a 30-min heat shock of the *hs*::*ΔNTbar-1* animals relative to *hs*::*control* animals.

We wished to identify Wnt pathway target genes in *C. elegans*, particularly those responsive to the “canonical” or WBC pathway, which is used at several points during larval development. Forward genetic approaches, although successful at identifying pathway components, have identified few Wnt responsive genes, and bioinformatic methods are of limited utility given the high number of possible POP-1 binding sites (YTTTGWW) in the *C. elegans* genome (>300,000 sites; K. Thompson and D. M. Eisenmann, unpublished results). Therefore, we chose microarray analysis of whole-worm transcriptomes to identify target genes responsive to Wnt pathway activation. One approach would be to compare gene expression in Wnt pathway mutants to that in wild-type animals; however, Wnt pathway inactivation causes changes in cell fate in several tissues during development (Eisenmann 2005; Jackson and Eisenmann 2012), and these alterations could affect our ability to characterize Wnt-dependent gene expression in cells of interest (such as the VPCs). Therefore, we compared gene expression in animals in which the Wnt pathway was conditionally activated to control animals in which it was not. To conditionally activate Wnt signaling, we used *hs*::*ΔNTbar-1*, a transgene that uses the heat shock promoter to express a BAR-1 protein with an amino-terminal deletion (ΔNTBAR-1) predicted to stabilize the protein in the absence of Wnt signaling (Figure 1A) (Gleason *et al.* 2002). A single heat shock of *hs*::*ΔNTbar-1* animals is sufficient to cause activated Wnt signaling phenotypes in fate specification of the ventral hypodermal VPCs and the lateral hypodermal seam cells (Gleason *et al.* 2002; Gleason and Eisenmann 2010). As a control, we used animals containing a transgene with the empty heat shock vector (*hs*::*control*).

We overexpressed ΔNTBAR-1 at the L2/L3 molt (24 hr posthatching), a time during larval development when we showed that both the VPCs and seam cells are susceptible to the activation of Wnt signaling (Gleason *et al.* 2002; Gleason and Eisenmann 2010). Synchronized populations of *hs*::*ΔNTbar-1* and *hs*::*control* worms were grown at 20° to the L2/L3 molt, given a single heat shock at 38° for 30 min, and returned to 20° for further development. This heat shock protocol generates >90% Muv animals, indicating robust pathway activation (Gleason *et al.* 2002). Following this single heat shock, ΔNTBAR-1 protein is present within 1 hr and peaks at 4 hr after heat shock (Figure 1B). To identify an optimal time for collection of RNA after heat shock, we examined the induction of the Hox gene *lin-39*, a putative Wnt target in the VPCs (Eisenmann *et al.* 1998; Wagmaister *et al.* 2006), and the LIN-39 target *egl-18*, which is likely to be an indirect Wnt pathway target in those cells (Koh *et al.* 2002). Based on these data (Figure 1C), and to bias the microarray analysis toward possible direct Wnt pathway target genes, we allowed heat-shocked animals to recover for only 60 min before collecting RNA. Whole worm gene expression patterns were determined for three independent biological replicates for experimental and control strains using the Affymetrix *C. elegans* genome microarray.

BAR-1 responsive genes were identified as those that were differentially expressed an average of two fold or more, with a *p*-value ≤ 0.05. These criteria revealed differential expression of 166 genes in response to expression of ΔNTBAR-1, corresponding to 0.8% of the transcripts on the array (Table S1). Of these, 104 genes were up-regulated, with an average increase of 3.3X (range 2.0–21.1X), whereas 62 genes were down-regulated, with an average decrease of 7.2X (range = 2.0–244.4X; however, all but one gene fell in the range 2.0–16.8X, with an average decrease of 3.5X). We propose that these ΔNTBAR-1−responsive genes are candidate Wnt pathway targets.

Interestingly, we noticed that almost one-quarter of the BAR-1 responsive genes were located near each other on the chromosome (41/166 genes; Table S2). Nineteen of the 104 up-regulated genes are on the same cosmid as another up-regulated gene, and almost one-third of the down-regulated genes (20/62) are near another down-regulated gene. Only one of these target gene pairs has been identified as an operon in previous analysis (*C08B11.7/9*) (Blumenthal *et al.* 2002). We calculated that if the entire 100 Mb *C. elegans* genome were contained in cosmids, the probability of 19 pairs of genes located on the same cosmid out of 104 genes is <10^−18^, suggesting that the observed clustering is nonrandom. One interpretation of this result is that the clustered genes may be co-regulated from common BAR-1 responsive enhancers located in their vicinity.

### Genes up-regulated upon conditional Wnt pathway activation

Of the 104 up-regulated genes, 51 have been studied previously, 15 encode proteins with an annotated activity, domain or homolog, and 38 are novel genes encoding unknown proteins (Table S1). The products encoded by known genes include collagen proteins (*bli-1*, *col-38*, *col-49*, *col-71*, *col-101*, *col-138*, *col-176*, *lon-3*), proteins involved in protein ubiquitylation and proteolysis (*fbxa-116*, *ubh-4*, *Y47D3B.11*, *F07A11.4*), putative targets of the DAF/insulin signaling pathway (*dao-4*, *dod-21*, *dod-23*), secreted proteins related to hedgehog ligands (*grl-10*, *hog-1*, *wrt-4*), nematode-specific peptides (*nspd-3*, *nspd-6*), O-acyl transferases (*oac-29*, *oac-30*, *oac-31*), UDP-glucuronosyltransferases (*ugt-8*, *ugt-19*), ADP-ribosylation factors (*arf-1.1*, *arl-7*), and aspartyl proteases (*F21F8.4*, *F59D6.3*) [reference for all genes is Wormbase (Harris *et al.* 2010; Yook *et al.* 2012)]. Gene Ontology term analysis indicates that the only statistically overrepresented gene class among the up-regulated genes was genes encoding structural components of the cuticle such as collagens (Table S3). Interestingly, unlike the known Wnt targets in *C. elegans*, only one of the 104 BAR-1 responsive up-regulated genes encodes a transcription factor (*sptf-2*); the category of nucleic-acid binding proteins was under-represented (Table S3).

Among the up-regulated genes, expression of the *pry-1/Axin* gene was increased 2.4-fold. Axin is a negative regulator of the canonical Wnt signaling pathway, and genes encoding Axin homologs are Wnt pathway targets in vertebrate cells and *Drosophila* (Jho *et al.* 2002; Lustig *et al.* 2002). The observed up-regulation of *pry-1* in response to activated BAR-1 expression indicates that negative pathway feedback via Axin gene up-regulation is likely to be an evolutionarily ancient mechanism of canonical Wnt signaling pathway modulation.

The known Wnt-regulated genes (WBA: *ceh-10*, *ceh-22*, *egl-18*, *elt-6*, *end-1*, *end-3*, and *psa-3*; WBC*: mab-5*, *egl-5*, and *lin-39)* did not meet the criteria for Wnt-regulated genes (Table S4). All of these genes, except *lin-39*, respond to Wnt signaling during the embryonic or early larval stages of development and might not be expected to be up-regulated in L3 larva. Additionally, HOX genes such as *lin-39* and *mab-5* are expressed in only a few cells and microarray analysis of total worm RNA may not be sensitive enough to detect changes in their levels. Consistent with this, the optimization experiment described above showed that *lin-39* levels do increase when assayed by qPCR. This result suggests that our application of stringent criteria may have identified those genes expressed in a larger number of cells and/or showing larger changes in transcript abundance.

### Genes down-regulated upon conditional Wnt pathway activation

Of the 62 down-regulated genes, 38 have been analyzed previously, four encode proteins with an annotated activity, domain or homolog, and 20 encode unknown proteins (Table S1). The products encoded by the down-regulated genes encode collagen proteins (*col-91*, *col-150*, *col-180*), nematode-specific peptides (*nspb-10*, *nspd-11*, *nspd-12*, *nspe-1*, *nspe-5*, *nspe-7*), P-glycoproteins (*pgp-6*, *pgp-14*) and UDP-glucuronosyltransferases (*ugt-29*, *ugt-54*) [reference for all genes is Wormbase (Harris *et al.* 2010; Yook *et al.* 2012)]. Several of these gene categories were also common among the list of up-regulated genes (collagen, *nsp*, and *ugt* genes). In addition, six of the genes down-regulated by ΔNTBAR -1 expression have been identified as up-regulated when the unfolded protein response is blocked (*abu-1*, *abu-6*, *abu-7*, *abu-8*, *abu-10*, *abu-11*) (Urano *et al.* 2002). No statistically overrepresented gene class was found among the down-regulated genes (Table S3).

Since Wnt pathway activation is almost universally known to act via direct up-regulation of target gene expression, we consider that the up-regulated genes we identified represent possible direct targets of Wnt signaling in *C. elegans*, whereas the down-regulated genes are more likely to be indirect targets. We did not pursue further analysis of the BAR-1 responsive down-regulated genes.

### Validation of BAR-1 responsive genes

To identify genes to characterize further, the regulation of these putative targets by Wnt signaling was validated by qPCR. As with the microarray experiments, we compared specific transcript levels between *hs*::*control* and *hs*::*ΔNTbar-1* animals heat-shocked at the L2/L3 molt; however, we also examined transcript levels in heat-shocked animals carrying the integrated transgene *hs*::*ΔNTpop-1*. *ΔNTpop-1* encodes a dominant negative variant of POP-1 in which the amino terminal beta-catenin binding domain is deleted (Korswagen *et al.* 2000). Expression of ΔNTPOP-1 causes a Wnt pathway reduction-of-function phenotype, or blocks expression of a Wnt hyperactive phenotype, in both neurons and hypodermal cells that rely on the WBC pathway during larval development (Korswagen *et al.* 2000; Gleason *et al.* 2002). We expected *bona fide* Wnt pathway targets to show one of two patterns of expression in this analysis. If the target gene is normally expressed at the L2/L3 molt, gene expression should increase with exposure to ΔNTBAR-1 and decrease in response to ΔNTPOP-1 (termed “class 1” targets). Alternatively, if the Wnt pathway target gene is not strongly expressed at the L2/L3 molt, we expect increased expression with exposure to ΔNTBAR-1 but little change in expression in response to ΔNTPOP-1 (termed “class 2” targets). We examined expression of 100 of the 104 up-regulated genes by qPCR and found 39 of 100 were reproducibly up-regulated twofold or greater after ΔNTBAR-1 overexpression in these experiments (data not shown). Of these, 13 were down-regulated 1.7-fold or more in response to expression of dominant negative ΔNTPOP-1 (class 1; Table 1), whereas nine were not (class 2; Table 1). The remaining BAR-1 responsive genes (17/39) were up-regulated upon both ΔNTBAR- and ΔNTPOP-1 overexpression, which is not expected for targets of the WBC or “canonical” Wnt pathway. We pursued further molecular and biological analysis of the 22 qPCR-validated, BAR-1 responsive genes.

**Table 1:** Validated Wnt target genes

		*ΔNTbar-1*	*ΔNTbar-1*	*ΔNTbar-1* *daf-16(lf)*	*ΔNTpop-1*			*bar-1(lf)*	*pry-1(lf)*		
Gene	Protein	MA	qPCR	qPCR	qPCR	DAF-16 dep	Class	qPCR	qPCR	Time	Expression Pattern
*bli-1*	Collagen	6.5	13.6	43.5	0.6	n	2	0.5	7.9	yAd	L4 peak
*col-38*	Collagen	4.8	12.7	62.2	0.3	n	1	0.2	2.8	yAd	L4 peak
*col-49*	Collagen	3.9	14.3	13.8	0.2	n	1	0.1	2.5	yAd	L4 peak
*col-71*	Collagen	3.6	14.5	36.9	0.6	n	2	0.2	3.2	yAd	L4 peak
*dao-4*	Novel	6.5	9.0	7.1	0.1	n	1	0.1	2.8	yAd	L4 peak
*F08G2.7*	Novel	2.7	4.7	10.7	0.7	n	2	0.4	2.5	L4	Flat
*oac-30*	O-acyltransferase	7.1	2.2	34.3	0.1	n	1	0.4	1.0	yAd	Oscillating
*pry-1*	Axin	2.4	3.2	39.5	0.7	n	2	0.3	2.1	L2	Flat
*sptf-2*	Zinc finger	2.5	3.0	3.5	0.2	n	1	0.3	2.6	yAd	Flat
*tag-164(ok771)*	Novel	4.9	2.8	20.4	0.3	n	1	0.3	3.7	yAd	Oscillating
*T26E4.4*	Novel	3.1	3.9	22.3	0.4	n	1	0.5	1.0	yAd	Oscillating
*Y41C4A.11*	Novel	20.6	2.5	3.2	1.0	n	2	0.3	2.6	yAd	L4 peak
*Y43C5A.3*	Novel	2.3	2.7	11.1	1.7	n	2	0.5	2.0	yAd	Flat
*Y71D11A.3*	Novel	4.6	2.7	7.8	1.6	n	2	0.5	1.0	L1	Flat
*arf-1.1*	ADP-ribosylation factor	21.1	14.6	0.1	0.3	y	1	nd	nd	nd	
*cgt-2*	Ceramide glucosyltransferases	2.5	3.3	1.1	1.4	y	2	nd	nd	nd	
*F15E11.15*	Novel	3.7	2.2	1.8	0.4	y	1	nd	nd	nd	
*oac-31*	*O*-acyltransferase	2.0	3.3	0.8	0.8	y	2	nd	nd	nd	
*E03H4.4*	Novel	2.3	26.6	307.1	0.1	n	1	0.1	*0.2*	L3	Oscillating
*fbxa-116*	F box protein	3.6	3.0	nd	0.3	nd	1	*1.0*	*0.1*	L1	Flat
*srw-93*	7TM receptor	3.3	3.3	74.5	0.5	n	1	*1.0*	*0.1*	L3	Flat
*dod-23*	Novel	3.3	4.6	129.8	0.1	n	1	4.5	1.0	yAd	L4 peak

Twenty-two genes that were up-regulated 60 min after expression of ΔNTBAR-1 at the L2/L3 molt are shown; gene name (column 1) and encoded protein function, if known (column 2), are shown. Columns 3 and 4 show the average fold change after ΔNTBAR-1 expression in microarray (MA) and qPCR experiments, respectively. Column 5 shows average fold change after ΔNTBAR-1 expression in a *daf-16* mutant background. Column 6 shows average fold change after ΔNTPOP-1 expression at a similar time point. Column 7 indicates if an increase in expression after exposure to ΔNTBAR-1 was dependent on DAF-16 function. In column 8, class 1 genes show increased expression with ΔNTBAR-1 and decreased expression with ΔNTPOP-1; class 2 genes show increased expression with ΔNTBAR-1 and no change with ΔNTPOP-1. Columns 9 and 10 show average fold change in strains with reduced Wnt signaling (*bar-1(ga80)*) or increased Wnt signaling (*pry-1(mu38)*), respectively; for each gene, the analysis was performed at the time point indicated in column 11. Column 12 shows expression pattern based on YFP reporter gene expression, as described in the text. Y, yes; n, no; nd, not determined; yAd, young adult. Average fold change is the average increase in expression of triplicate experiments; experiments performed by heat shock expression at the L2/L3 molt (columns 3− 6) were compared with heat shock control animals; experiment with *bar-1* and *pry-1* mutant strains (columns 9 and 10) were compared with N2 control animals. MA, microarray; qRT-PCR, quantitative reverse transcription polymerase chain reaction.

### A subset of BAR-1 responsive genes are DAF-16 dependent

In addition to interacting with POP-1/TCF, under conditions of oxidative stress, BAR-1 interacts with the FOXO transcription factor DAF-16 and can increase target gene expression (Essers *et al.* 2005). Therefore, some of our BAR-1 responsive genes could be targets of a BAR-1/DAF-16 interaction. To address this, we compared transcript abundance by qPCR in heat shocked *hs*:: Δ*NT-bar-1* animals and *hs*:: Δ*NT-bar-1*; *daf-16(mgDf50)* animals. We found that four of the 22 BAR-1 responsive genes (*arf-1.1*, *cgt-2*, *F15E11.15*, *oac-31*) were no longer strongly up-regulated by ΔNTBAR-1 in the absence of DAF-16 (Table 1). Interestingly, we noted an increase in expression for 12 of the other 18 BAR-1 responsive target genes in the *hs*:: Δ*NT-bar-1*; *daf-16(mgDf50)* background compared with *hs*:: Δ*NT-bar-1* alone. One possibility is that loss of DAF-16 may increase the pool of BAR-1 available for interaction with POP-1 at the regulatory regions of Wnt target genes, increasing their activation even further. Curiously, the gene *dao-4*, which was identified as a downstream target of the DAF-2/DAF-16 pathway previously (Jiang *et al.* 2001), did not display DAF-16 dependence for its up-regulation by BAR-1. It is possible that *dao-4* is a target of both Wnt (POP-1/BAR-1) and insulin (DAF-16/BAR-1) signaling. Further analysis of the four DAF-16−dependent, BAR-1− responsive genes was not pursued.

### Target genes showed altered expression in Wnt pathway mutant backgrounds *in vivo*

We examined expression of the remaining 18 BAR-1 responsive genes in two Wnt pathway mutant backgrounds. To decrease “canonical” Wnt signaling, we used a *bar-1(ga80)* strain, which contains a putative null mutation affecting BAR-1/beta-catenin. *bar-1(ga80)* animals have a number of developmental defects and reduced expression of the putative Wnt target genes *lin-39*, *mab-5*, and *egl-5* (Eisenmann *et al.* 1998; Maloof *et al.* 1999; Gleason *et al.* 2002; Korswagen *et al.* 2002). To increase “canonical” Wnt signaling, we used a *pry-1(mu38)* strain, which contains a loss-of-function mutation affecting the Wnt pathway negative regulator PRY-1/Axin. *pry-1(mu38)* animals display ectopic expression of the putative Wnt pathway targets *lin-39*, *mab-5*, and *egl-5*, whereas *pry-1* gain-of-function leads to reduced levels of these genes (Eisenmann *et al.* 1998; Maloof *et al.* 1999; Gleason *et al.* 2002; Howard and Sundaram 2002; Korswagen *et al.* 2002; Gleason *et al.* 2006; Oosterveen *et al.* 2007). qPCR analysis showed that fifteen of the eighteen genes analyzed showed decreased expression in the *bar-1(ga80)* background, and 11 of 18 BAR-1 responsive genes showed increased expression in a *pry-1(mu38)* background (Table 1). Four genes (*E03H4.4*, *srw-93*, *fbxa-116*, *dod*-23) did not display the behavior predicted for a Wnt target gene in these backgrounds.

In summary, 14 genes display increased expression in response to the conditional expression of ΔNTBAR-1 and show altered expression in *bar-1(ga80)* or *pry-1(mu38)* mutant backgrounds. These genes (*bli-1*, *col-38*, *col-49*, *col-71*, *dao-4*, *F08G2.7*, *oac-30*, *pry-1*, *sptf-2*, *tag-164(ok771)*, *T26E4.4*, *Y41C4A.11*, *Y43C5A.3*, *Y71D11A.3*) are the strongest candidates among the BAR-1 responsive genes for being *bona fide* Wnt pathway target genes. The products of these genes include four collagens, the Wnt pathway component PRY-1/Axin, an O-acyltransferase, a Zinc-finger transcription factor, and seven novel proteins (Table 1).

### Temporal and spatial expression of Wnt pathway responsive genes

**Figure 2:**
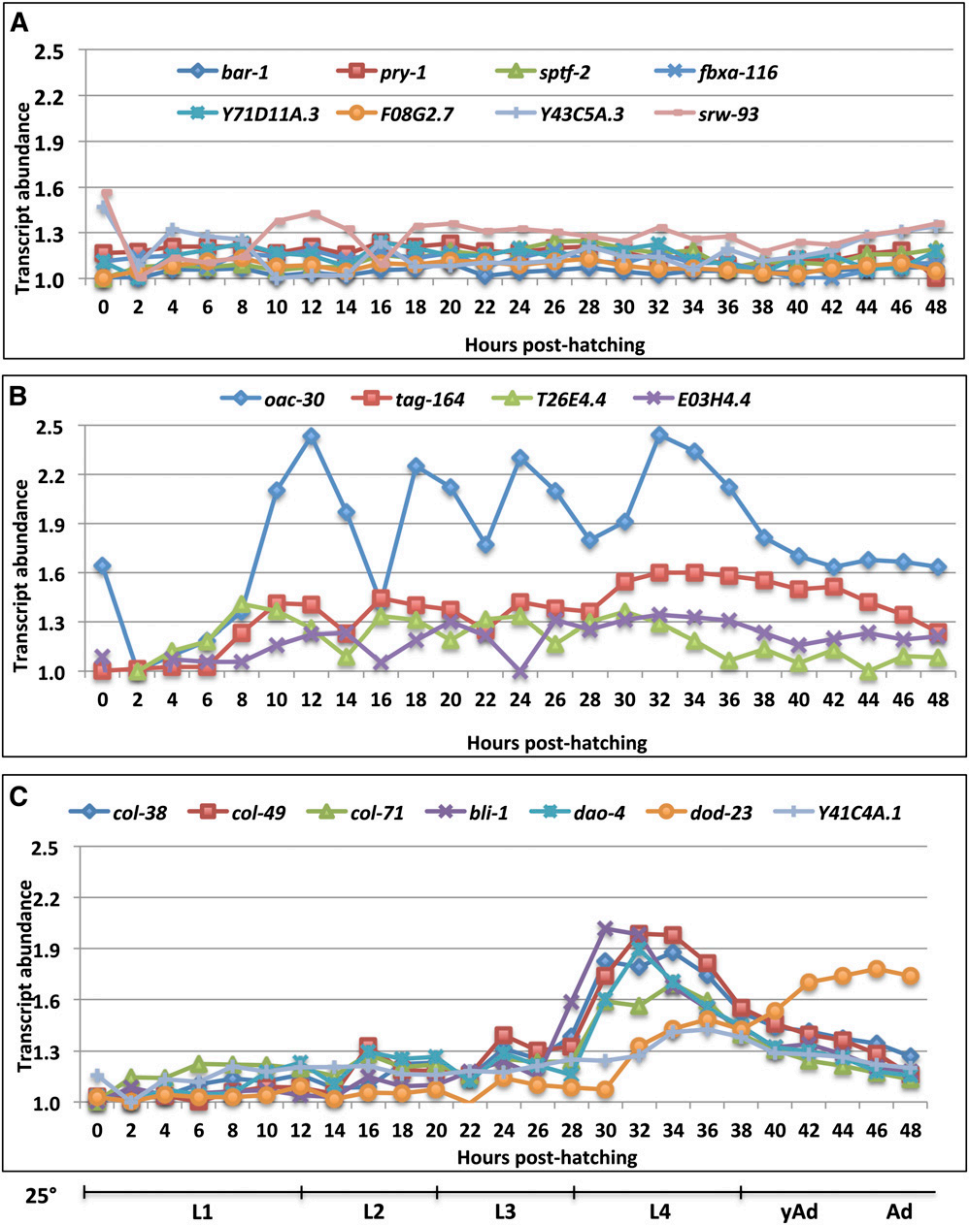
Temporal expression of BAR-1 responsive genes during larval development. Quantitative polymerase chain reaction analysis of selected BAR-1 responsive genes from wild-type animals grown at 25°. RNA samples were taken every 2 hr from hatching to gravid adulthood. For each gene, the data were normalized to the lowest expression point, which was assigned a value of 1.0. The approximate extent of each developmental stage is indicated below the figure. (A) eight genes showing a constant expression pattern during development; (B) four genes showing a peak of expression in each larval stage; (C) seven genes showing a peak of expression in the L4 stage.

To determine the normal temporal pattern of expression of these Wnt responsive genes, we examined their transcript levels every 2 hr during wild-type development from the L1 to adult stages by qPCR (see Johnstone and Barry 1996); we observed three patterns of expression. First, a flat pattern of expression with little or no change across developmental time was seen for *fbxa-116*, *F08G2.7*, *pry-1*, *sptf-2*, *srw-93*, *Y43C5A.3*, and *Y71D11A.3* (Figure 2A). Second, an oscillating expression pattern, with a peak observed in each of the four larval stages was observed for *E03H4.4*, *oac-30*, *tag-164(ok771)*, and *T26E4.4* (Figure 2B). This type of oscillating expression during larval life has been observed for collagen genes involved in cuticle synthesis, which occurs once in each larval stage (Johnstone and Barry 1996). Third, a distinct peak in expression in the L4 stage was observed for *bli-1*, *col-38*, *col-49*, *col-71*, *dao-4*, *dod-23*, and *Y41C4A.11* (Figure 2C). This pattern is interesting because it indicates that these genes, although normally showing a peak of expression in the L4 stage, can be precociously expressed at the L3 stage in response to ΔNTBAR-1 expression. It is noteworthy that four of the seven “L4 peak” genes encode cuticle collagens. Cuticle collagens are small, collagen-like proteins that are the major component of the *C. elegans* cuticle (Kramer 1997; Johnstone 2000; Myllyharju and Kivirikko 2004). Previously, several other collagen genes have been shown to be expressed at the time of the L4/adult molt, suggesting they are used in the formation of the adult cuticle (Cox and Hirsh 1985; Liu *et al.* 1995).

**Figure 3:**
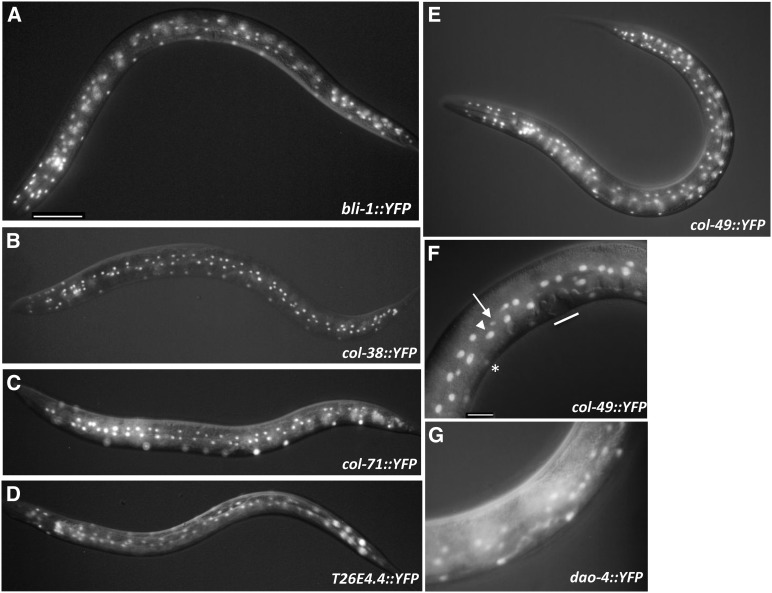
L4 expressed Wnt target genes are expressed in hyp7 hypodermal syncytium. Mid**-**L4 stage animals showing expression of indicated YFP transcriptional reporter in nuclei of the hyp7 syncytial hypodermis, including cells generated by division of the lateral seam cells and the ventral P cells. Each figure shows overlapping fluorescence and Nomarski images. (A) *bli-1*::*YFP*; (B) *col-38*::*YFP*; (C) *col-71*::*YFP*; (D) *T26E4.4*::*YFP*; (E) *col-49*::*YFP*; (F) *col-49*::*YFP*, close-up showing expression in seam cells (arrow), hypodermal daughters of seam cells (arrowhead), daughters of nonvulval Pn.p cells (asterisk), but not in vulval cells (line); (G) *dao-4*::*YFP*. Scale bar in (A) and (F) = 50 um.

To determine the spatial expression patterns of these genes, we created transcriptional reporters for 10 of these genes (*bli-1*, *col-38*, *col-49*, *col-71*, *dao-4*, *pry-1*, *tag-164(ok771)*, *T26E4.4*, *Y43C5A*.3, and *Y41C4A.11)*; these reporters contained the DNA upstream of the gene of interest fused to YFP coding sequences (Table S5). The upstream region of all genes but *col-71*, *Y41C4A.11*, and *tag-164(ok771)* was sufficient to drive YFP expression. For *col-71* and *Y41C4A.11*, the inclusion of large first and second introns allowed expression to be observed; expression was never observed for *tag-164(ok771)*::*YFP*. Expression for *bli-1_(p)_*::*YFP*, *col-38_(p)_*::*YFP*, *col-49_(p)_*::*YFP*, *col-71_(p)_*::*YFP* and *dao-4_(p)_*::*YFP* was seen in nuclei of the hyp7 syncytial hypodermis in the L4 and young adult stages, including nuclei generated from divisions of lateral seam cells and ventral P cells (Figure 3). Expression was also seen in the seam cells themselves in the L4 stage, but not in the cells of the developing vulva (Figure 3). YFP expression was also observed in hyp7 for *T26E4.4_(p)_*::*YFP* and *Y43C5A.3_(p)_*::*YFP* but was seen as early as the L2 stage (Figure 3 and data not shown). We previously showed that *bar-1* is expressed in these same hypodermal cells/nuclei at this time (Eisenmann *et al.* 1998; Natarajan *et al.* 2004). As previously described (Korswagen *et al.* 2002), *pry-1_(p)_*::*YFP* expression was seen in numerous cells in the embryo, and in the seam cells, P cells, VPC, and ventral cord neurons during all larval stages (Figure S1, A and B). Expression of *Y41C4A.11_(p)_*::*YFP* was observed in cells of the pharynx from the L1 through L4 stages (Figure S1C). The expression of an existing *sptf-2*::*GFP* transcriptional reporter in several head neurons was as described (Reece-Hoyes *et al.* 2007) (Figure S1D). Therefore, the YFP reporter data supports the developmental expression data and indicates that a small group of Wnt-responsive genes (*bli-1*, *col-38*, *col-49*, *col-71*, *dao-4*, *T26E4.4*, *Y43C5A.3)*, including several collagen-encoding genes, are expressed during normal development in hypodermal cells at the L4 larval stage.

### Wnt signaling regulates the temporal but not spatial expression of several *col* genes

Although these genes show increased expression when the Wnt pathway is activated with either a *pry-1/Axin* loss-of-function mutation or expression of ΔNTBAR-1 protein, it is not clear how the increase in expression of these genes is manifested. For example, Wnt pathway activation at the L2/L3 molt could induce expression in cells that do not normally express the genes, or could increase expression in cells that normally have low expression at this time. To examine this question, YFP reporters for three collagen genes that normally show peak expression in the L4 stage, *col-38*, *col-49*, and *bli-1*, were crossed into *hs*::*ΔNTbar-1*, *hs*::*ΔNTpop-1* and *hs*::*control* backgrounds and analyzed for changes in expression after heat shock. For all three *col* gene reporters, we observed an increase in the percentage of animals showing YFP expression in the L3 stage following heat shock expression of ΔNTBAR-1 at the L2/L3 molt, and a decrease in the percentage of animals showing YFP expression at all stages following heat shock expression of dominant negative ΔNTPOP-1 (Table 2). In all cases the YFP expression pattern was the same as that observed in wild type animals, only the penetrance of expression changed (data not shown). These results show that stimulating the Wnt signaling pathway alters the temporal but not the spatial pattern of expression for these three *col* genes, resulting in their expression earlier than their normal peak expression in the L4. This suggests that the normal function of Wnt signaling may be to contribute to expression of these genes in the hypodermal cells in the L4 stage, but that they are in a Wnt-sensitive state at earlier stages.

**Table 2:** Wnt signaling regulates the temporal but not spatial expression of several *col* genes

	L3		L4		Adult	
	n	%YFP^+^	n	%YFP^+^	n	%YFP^+^
*col-38p*::*YFP*						
* hs*::*control*	234	41%	188	82%	83	100%
* hs*::*ΔNTbar-1*	133	76%**	64	73%	68	66%**
* hs*::*ΔNTpop-1*	228	20%**	70	57%**	55	100%
*col-49p*::*YFP*						
* hs*::*control*	105	24%	194	80%	59	92%
* hs*::*ΔNTbar-1*	195	42%*	72	82%	56	82%
* hs*::*ΔNTpop-1*	118	0%**	126	29%**	51	96%
*bli-1p*::*YFP*						
* hs*::*control*	55	0%	45	93%	54	100%
* hs*::*ΔNTbar-1*	114	18%**	52	98%	99	39%**
* hs*::*ΔNTpop-1*	82	0%**	77	100%	72	92%

Arrays containing transcriptional reporters *col-38p*::*YFP*, *col-49p*::*YFP*, and *bli-1*::*YFP* were crossed into strains carrying the integrated arrays *hs*::*control*, *hs*::Δ*NTbar-1*, and *hs*::Δ*NTpop-1*. Each of the resulting nine strains was developmentally synchronized by starvation and L1 feeding, then given a heat shock at the L2/L3 molt. YFP expression was scored at the L3 (columns 2 and 3; 25 hr postfeeding), L4 (columns 4 and 5; 40 hr postfeeding), and young adult stages (columns 6 and 7; 50 hr postfeeding). The number of animals scored and percent of animals showing any YFP expression in hypodermal cells is indicated.

* *P* < 0.05; **P < 0.0001 (Fisher’s exact t-test) significant difference in expression compared with the *hs*::*control* strain

### A small subset of collagen genes are expressed in the L4 and are Wnt-responsive

**Figure 4:**
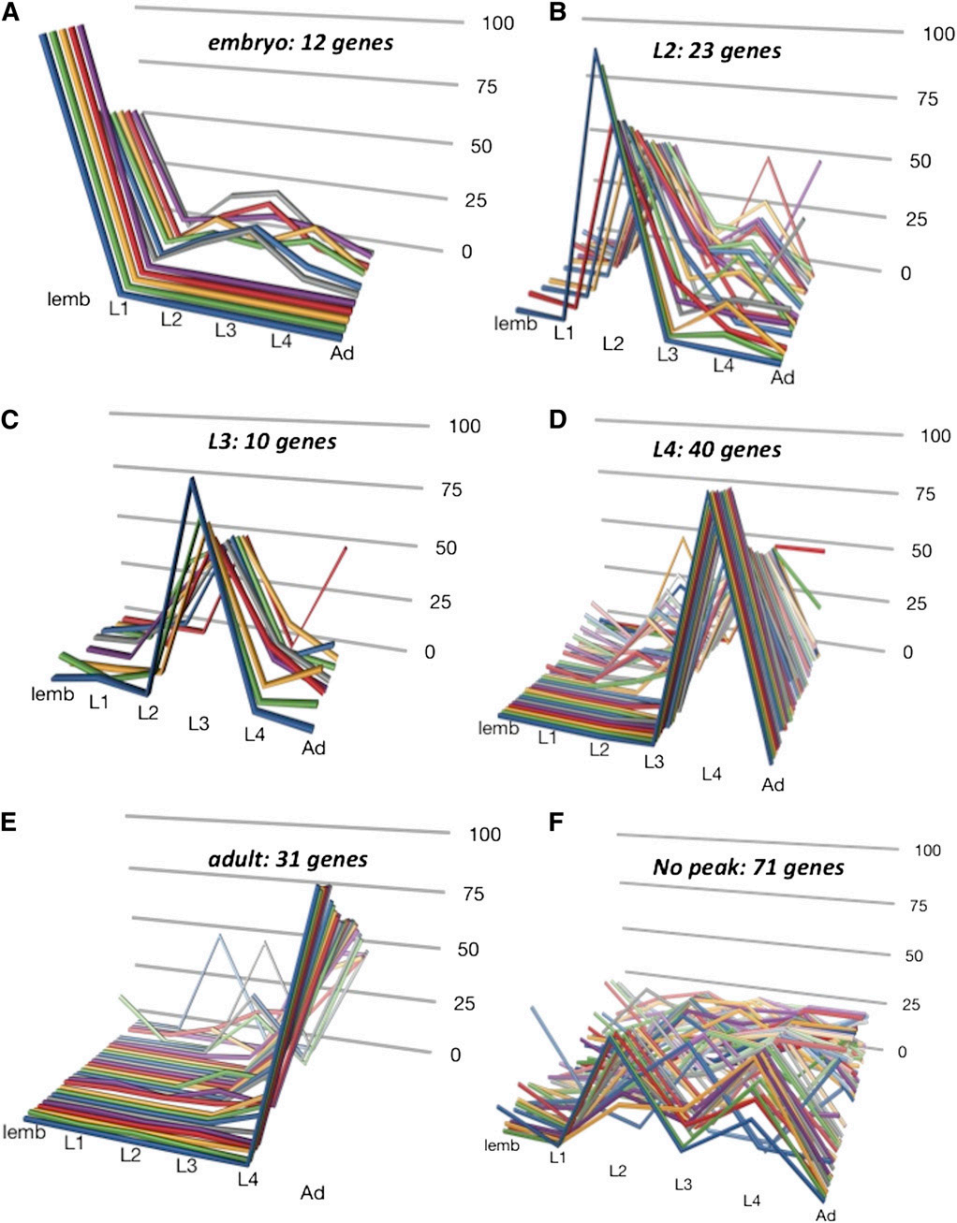
*col* gene peak expression data. Expression data for each *C. elegans* gene encoding a protein with a collagen motif were extracted from the modENDCODE project development expression series (Gerstein *et al.* 2010) and those genes showing 50% or more of their total expression in a particular stage of development were identified (see File S1 and Table S6). For each graph, 100% indicates the total of expression across six developmental time points (late embryo, L1, L2, L3, L4 adult) and the percent expression of each gene in each stage is graphed. “Peak” expression is defined as ≥50% of total expression in any one stage. (A) 10 genes with peak expression in the late embryo; (B) 23 genes with peak expression in the L2 stage; (C) 10 genes with peak expression in the L3 stage; (D) 40 genes with peak expression in the L4 stage; (E) 31 genes with peak expression in the adult; and (F) 71 genes without a peak of expression, as defined previously. Note, only one gene shows peak expression in the L1 stage (not shown).

We wanted to know whether all *col* genes showing L4-specific expression were identified in our microarray analysis, or if only a subset of *col* genes showing peak expression in the L4 were found as Wnt pathway targets. There are 188 *C. elegans* genes that encoded proteins annotated with a collagen or cuticle collagen protein motif in the *C. elegans* database (Harris *et al.* 2010; Yook *et al.* 2012) (we collectively refer to these as *col* genes, even though not all gene names begin with *col*) (Kramer 1994; Johnstone 2000; Myllyharju and Kivirikko 2004). The vast majority of these genes (164) encode nematode cuticle collagens: a small, novel type of collagen found as a major constituent of the nematode cuticle at all stages (Johnstone 2000; Myllyharju and Kivirikko 2004). Recently, the modENCODE project carried out RNAseq analysis of all *C. elegans* genes across seven developmental time points from the early embryo to young adult stages, as well as during entry and exit from the dauer larva alternative developmental stage (Gerstein *et al.* 2010). We examined these data to (1) determine the developmental expression patterns of all *col* genes, and (2) to identify genes with peak expression in the L4 stage. Previous molecular analysis has shown that some *col* genes are expressed at all larval stages, whereas others show increased expression in particular larval stages or in animals entering the dauer stage (Cox and Hirsh 1985; Kramer *et al.* 1985; Levy and Kramer 1993; Liu *et al.* 1995; Johnstone and Barry 1996). Consistent with these earlier results, we found that 117 of the *col* genes analyzed (62%) showed more than half of their total developmental expression during only one stage. Another 28 *col* genes (15%) showed more expression in the dauer larva than in all other normal developmental stages combined. We categorized the *col* genes by the stage at which they showed the majority of their expression, *e.g.*, “L3-peak” (Figure 4, File S1 and Table S6). The modENCODE data support our development time course data; *bli-1*, *col-38*, *col-49*, and *col-71* are all identified as “L4-peak” genes. Three points stand out from this analysis. First, most *col* genes may not show cyclic expression in each of the four larval stages as was observed for a number of *col* genes (Johnstone and Barry 1996) but show a majority of their expression in one developmental stage, as seen for other *col* genes (Cox and Hirsh 1985; Kramer *et al.* 1985; Levy and Kramer 1993; Liu *et al.* 1995). Second, no *col* genes with peak expression in the embryo, L1, L2, or dauer larva were found as BAR-1 responsive genes by the microarray analysis, suggesting these genes are not sensitive to ectopic Wnt signaling at the L2/L3 molt (Table S1). Third, the four “L4-peak” *col* genes we identified as Wnt pathway responsive genes represent only 10% of 40 “L4-peak” *col* genes, suggesting that a specific subset of *col* genes expressed preferentially in the L4 larva may be regulated by Wnt signaling at that time.

To identify other genes that may function in a common process with these Wnt-responsive, “L4 peak” *col* genes, we asked which other BAR-1 responsive genes were also expressed preferentially in the L4 stage. Analysis of the modENCODE developmental data set in the same manner as for the *col* genes identified 794 genes that show ≥50% of their total developmental expression in the L4 stage (File S2 and Table S7), of which only 12 were identified as BAR-1 responsive genes by our microarray analysis (*bli-1*, *col-38*, *col-49*, *col-71*, *col-138*, *dao-4*, *nspd-3*, *tag-164(ok771)*, *H23N18.5*, *Y41C4A.11*, *Y69E1A.2*, and *ZK180.6)*. These data indicate that the majority of the BAR-1 responsive genes do not have a peak of expression in the L4 stage. However, the similar temporal expression of a small subset of collagen-encoding genes (*bli-1*, *col-38*, *col-49*, *col-71*, *col-138*) and several novel genes (*dao-4*, *nspd-3*, *tag-164(ok771)*, *H23N18.5*, *Y41C4A.11*, *Y69E1A.2*, and *ZK180.6)* suggests that these genes may function in a common Wnt-regulated process at the L4 stage, perhaps involving adult cuticle synthesis.

### RNAi knockdown of several BAR-1 responsive genes causes body morphology, molting, vulval induction, and other phenotypes

Except for *bli-1* (Brenner 1974), none of the *col* genes we identified as Wnt pathway targets has been mutated previously. To begin to address the biological function of these *col* genes and the other genes with similar temporal and spatial expression patterns, we used RNA interference to reduce their expression and assayed for any obvious phenotypes. We used the method of “L1 feeding RNAi”: wild-type animals were synchronized as L1 larvae and grown at 15°, 20°, and 25° on *E. coli* expressing dsRNA specific to each gene of interest (Ahringer 2006). These same animals were then examined at the L3, L4, and young adult stages for phenotypes associated with Wnt signaling defects, known collagen mutant phenotypes, molting, or cuticle defects. We observed several defects suggesting that reduced function for some of these genes affects the integrity of the worm cuticle (Table 3).

**Table 3:** RNAi phenotypes

RNAi	Rupture Ad	Shrinkwrap Ad	Blister Ad	Dumpy Ad	Molt/Hyp L4	Molt/Hyp Ad	Vulva UI L4	Vulva OI Ad
Control	1	0	0	0	3	9	3	0
*col-38*	2	20**	0	3	24**	15	5	0
*col-49*	5	0	0	11*	3	7	9	0
*col-71*	4	0	0	8*	6	14*	3	0
*bli-1*	11*	0	97**	0	3	0	0	0
*dao-4*	3	0	0	9*^,^*^a^*	4	1	14*	0
*Y41C4A.11*	0	0	0	0	1	0	0	0
*sptf-2*	23**	5	0	0	5	3	3	0
*pry-1*	12*	0	0	0	7	23**	1	5*
*F08G2.7*	2	0	0	0	9*	12	4	0
*tag-164(ok771)*	3	0	0	0	23**	33**	5	0

Control RNAi animals (vector control) or animals treated as newly-hatched L1 larvae with RNAi to reduce function of the indicated gene were examined for several phenotypes (indicated in the text) as adults (Ad) or in the midL4 stage (L4). RNAi, RNA interference.

* *P* < 0.05, **P < 0.001, Fisher t-test. N > 25 animals scored for all data

#### Body morphology phenotypes:

**Figure 5:**
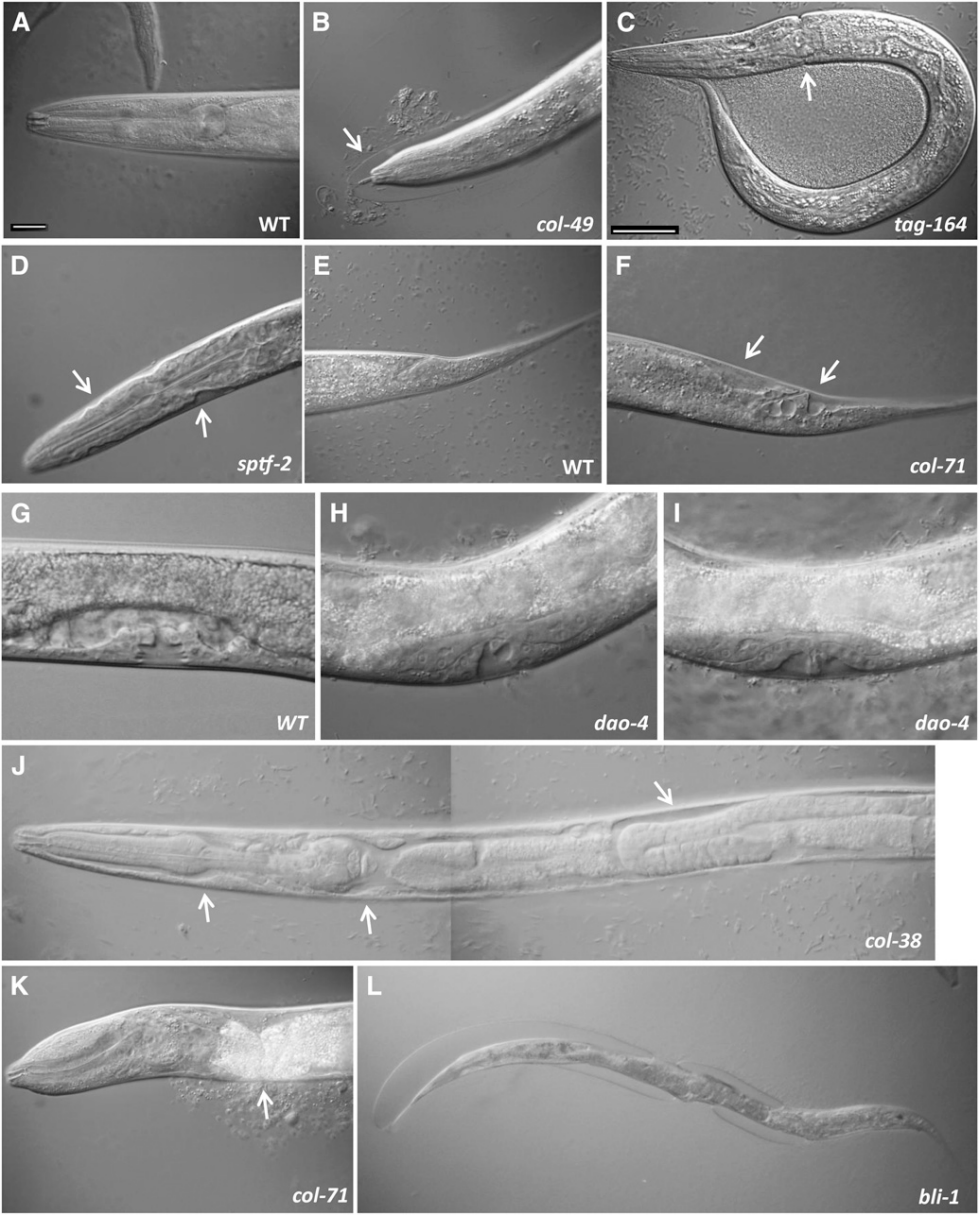
RNAi phenotypes of selected BAR-1 responsive genes. Control RNAi animals (WT = vector control) or animals treated as newly-hatched L1 larvae with RNAi to reduce function of the indicated gene are shown. Animals in (A–K) are mid-L4 stage hermaphrodites. (A) Head region of WT animal; (B) head region of *col-49(RNAi)* animal showing undetached cuticle (arrow); (C) *tag-64(RNAi)* animal showing ring constriction from undetached cuticle (arrow); (D) head region of *sptf-2(RNAi)* animal showing regions where hypodermal cells have retracted from overlying cuticle (arrows); (E) tail region of WT animal; (F) tail region of *col-71(RNAi)* animal showing regions in which hypodermal cells have retracted from overlying cuticle (arrows); (G) vulval invagination (“Christmas tree” stage) in WT animal; (H–I) defective vulval invaginations in *dao-4(RNAi)* animals; (J) anterior half of body of *col-38(RNAi)* animal showing regions in which internal organs appear to have “shrunk” within the psuedocoelomic cavity (arrows; compare to morphology in all other panels); (K) *col-71(RNAi)* animal showing Rupture phenotype at an anterior ventral location (arrow); (L) Bli phenotype in adult *bli-1(RNAi)* hermaphrodite. Scale bar in (A) and (C) = 50 μm. RNAi, RNA interference; WT, wild type.

Most previously described mutations in collagen genes affect body size or morphology (Myllyharju and Kivirikko 2004; Page and Johnstone 2007), as expected for proteins that are the major component of the cuticle, the structure that gives the worm its shape (Kramer 1997; Page and Johnstone 2007). Known mutants display Dumpy, Squat, and Long body size phenotypes, but also Roller (altered mobility) and Blister phenotypes (accumulations of fluid in the cuticle). The *bli-1* gene is known to cause a Blister phenotype when mutated (Brenner 1974), and we observed this phenotype robustly with feeding RNAi treatment (Table 3 and Figure 5). We also observed a low penetrance Dumpy phenotype after RNAi for the collagen genes *col-49* and *col-71*, as well as for *dao-4*, which encodes an unknown protein. No other genes displayed body size/morphology phenotypes.

#### Molting/cuticle/hypodermis phenotypes:

*C. elegans* hypodermal cells secrete a cuticle layer in the embryo for use in the newly hatched L1 larva, and secrete a new cuticle at the end of each larval stage for use in the next stage (Kramer 1997; Page and Johnstone 2007). The cuticle molting cycle consists of three steps: the secretion of cuticle components by the underlying hypodermal cells and formation of a new cuticle under the old one; the separation of the old cuticle from the hypodermal cells; and shedding and escape from the old cuticle by stereotyped twisting motions (Singh and Sulston 1978). We observed two phenotypes that suggest a defect in the underlying cells that secrete the cuticle or in the molting process (Table 3). First, we observed animals with an Mlt phenotype (Frand *et al.* 2005), in which the old cuticle appeared to remain attached, usually at the head or tail, and sometimes manifest as a ring of cuticle around the head of the animal (Figure 5). Second, we observed animals in which the underlying hypodermal cells appeared to pull away from the overlying cuticle (Figure 5). We observed these phenotypes in animals treated to reduce function of *col-38*, *col-71*, *pry-1*, *tag-164(ok771)*, and *F08G2.7* at higher penetrance than control.

#### “Rupture” phenotype:

In *C. elegans*, internal hydrostatic pressure acting against the cuticle exoskeleton gives the worm its shape and allows locomotion (Kramer 1997; Page and Johnstone 2007). Breaks or weak points in the hypodermis or cuticle can result in the internal organs spewing out, typically at the vulval opening, referred to as a ‘Rupture’ phenotype. Animals treated by RNAi for *bli-1*, *sptf-2*, and *pry-1* displayed a Rupture phenotype at higher penetrance than control (Table 3; Figure 5). We also saw an increased frequency of Rupture when adult *col-71(RNAi)* worms were placed in distilled water (C. Kang and D. Eisenmann, unpublished results). The site consistently affected by the *col-38*, *col-71*, and *sptf-2* RNA was in the anterior region, not at the site of the vulva.

#### “Shrink wrap” phenotype:

Typically, in worms observed under Nomarski optics in M9 media, the internal organs of the worm fill the body cavity, and little “empty” psuedocoelomic space is evident. We observed a phenotype in *col-38* and *sptf-2* RNAi-treated animals that we refer to as “Shrink wrap” (Table 3). In affected animals there was a noticeable gap between the outer layer of hypodermis and muscle and the internal organs (intestine, gonad, and pharynx; Figure 5). “Shrink wrap” animals were observed to stop pharyngeal pumping and become immobile after a short time in M9 + levamisole: this behavior was not observed in the control animals. We do not know the cellular or physiological basis of this phenotype, but it could be due to defects in osmoregulation in slide mounting media.

#### Vulval underinduction phenotype:

During induction of the *C. elegans* vulva, the BAR-1 dependent Wnt signaling pathway functions in cell fate specification of the VPCs (Eisenmann *et al.* 1998). Overactivation of the pathway by expression of ΔNTBAR-1 or with a *pry-1/Axin* loss-of-function mutation results in an Overinduced (OI) phenotype (Gleason *et al.* 2002). We recapitulated the *pry-1* Overinduced phenotype weakly by RNAi (Table 3). Conversely, when Wnt signaling is compromised, too few VPCs adopt vulval fates resulting in an Underinduced (UI) phenotype. We found that RNAi treatment to reduce function of *dao-4* caused animals to display an Underinduced phenotype, similar to that seen in *bar-1(ga80)* mutant animals (Table 3 and Figure 5). This suggests that this gene, which is expressed in the hypodermis, seam cells and P cells (which give rise to the VPCs), may function downstream of Wnt signaling during vulval induction.

### RNAi knockdown of several BAR-1 responsive genes causes defects in cuticle integrity

The spatial and temporal expression pattern of *bli-1*, *col-38*, *col-49*, *col-71*, *dao-4*, *T26E4.4*, and *Y43C5A.3* suggests they may be used in the synthesis of the adult cuticle. The cuticle of *C. elegans* serves a barrier or protective function and renders the organism partially resistant to many pharmacological or chemical agents (Kramer 1997; Moribe *et al.* 2004; Partridge *et al.* 2008). Mutants with defects in cuticle integrity show hypersensitivity upon exposure to such compounds (Moribe *et al.* 2004; Partridge *et al.* 2008). To determine whether animals with reduced function for these Wnt-responsive genes showed defects in adult cuticle integrity or function, we exposed adult animals in liquid to the dye Hoechst 33258, which stains chromatin in animals with defective cuticle integrity (Moribe *et al.* 2004). We found that reduction of function for the collagen genes *bli-1*, *col-38*, and *col-49*, as well as the novel gene *dao-4*, caused adult animals to display increased permeability to Hoechst stain (Table 4 and Figure S2). Interestingly, *bar-1(ga80)* mutant animals, which have reduced canonical Wnt signaling, also showed this latter phenotype. These results suggest that compromising expression of these L4-expressed, BAR-1 responsive collagen genes and several other genes showing a similar temporal and spatial expression pattern, partially compromises function of the adult cuticle. This result is consistent with the model that these genes are expressed in the L4 stage for use in the synthesis or function of the adult cuticle.

**Table 4:** Cuticle permeability assay

Strain	Hoechst % Stained
N2	5%
*bar-1(ga80)*	25%**
*bli-1(e769)*	54%**
*tag-164(ok771)*	8%
*sptf-2(tm1130)*	6%
*vector control (RNAi)*	4%
*col-38(RNAi)*	17%**
*col-49(RNAi)*	25%**
*col-71(RNAi)*	7%
*dao-4(RNAi)*	15%**

Cuticle permeability was tested by a 15-min exposure to Hoechst dye in control animals (N2, vector control), animals carrying a *bar-1* loss-of-function mutation, or animals with reduced function for Wnt target genes (mutants for *bli-1*, *tag-164(ok771)*, *and sptf-2*; RNAi treatment for *col-38*, *col-49*, *col-17*, and *dao-4*). The percent of animals with significant nuclear staining after a 15-min treatment is indicated. RNAi, RNA interference.

** *P* < 0.0001, Fisher t-test

## Discussion

The beta-catenin dependent or canonical Wnt pathway plays a major role in the development and homeostasis of all metazoans (Clevers 2006; Cadigan and Peifer 2009; Macdonald *et al.* 2009; Clevers and Nusse 2012). The nematode *C. elegans* contains two beta-catenin dependent Wnt signaling pathways, one of which, termed the “Wnt BAR-1 canonical” pathway (WBC), is similar in components and mechanism to the pathway that has been characterized in Drosophila and vertebrates (Jackson and Eisenmann 2012). The *C. elegans* WBC pathway functions at several points during larval development, including during fate specification by several ventral epithelial cells, including the six VPCs (P3.p−P8.p) and two posterior cells (P11.p and P12.p). There are only three known WBC pathway targets in *C. elegans*, the Hox genes *lin-39*, *mab*-5, and *egl-5*, although in none of these cases has POP-1 binding been shown to be necessary for regulation (Eisenmann *et al.* 1998; Jiang and Sternberg 1998; Maloof *et al.* 1999). Our goal in this work was to use a genomic approach to identify WBC pathway target genes during *C. elegans* larval development. We conditionally activated the Wnt pathway during a defined stage in larval development by expressing a gain-of-function, truncated version of BAR-1 beta catenin from the heat shock promoter. We chose the L2/L3 molt, since previous analysis from our lab has shown that the VPCs are sensitive to expression of this reagent at this time, displaying a Wnt gain-of-function phenotype (Gleason *et al.* 2002). We collected RNA from experiment and control strains 60 min after the heat shock, and used these samples for microarray analysis to identify genes differentially regulated upon Wnt activation. The advantages of this method are (1) it circumvents any cell fate changes that might arise in the cells of interest that would occur if we used Wnt mutant strains; (2) it analyzes expression in the context of the whole organism during normal development unlike other methods which look at gene expression in early embryos or dissociated cells; and (3) due to the short time between the heat shock and RNA sample collection, it is likely to enrich for early targets activated by the pathway, which are more likely to be direct. This method has the caveat that ectopic expression of the activated BAR-1 may increase expression of genes that otherwise would not be expressed at that time, or may allow expression in ectopic locations. However, it should be noted that beta-catenin proteins do not bind DNA on their own but interact with a TCF partner that is pre-bound at promoters, which suggests that this method may only affect genes that are normally regulated by Wnt signaling.

Using this method and the selection criteria we applied to the microarray data, we identified 166 genes differentially regulated by expression of truncated BAR-1/beta-catenin. A total of 104 of these genes were up-regulated, as expected for Wnt pathway direct targets. Among the up-regulated genes are (1) a large number encoding enzymes involved in metabolism, signaling or proteolysis; (2) two encoding proteins containing hedgehog-related domains; (3) three genes identified as downstream targets of the dauer/insulin signaling pathway; (4) eight genes encoding components of the cuticle; and (5) a large number of genes encoding proteins of unknown function. Interestingly, unlike the Wnt target genes identified by previous genetic analysis of Wnt dependent processes, only one of our genes up-regulated upon Wnt pathway activation encodes a transcription factor.

We narrowed our focus for further characterization of select target genes using a series of assays. First, we validated 39 of 100 genes as up-regulated 1 hr after expression of the dominant activated beta-catenin protein using qPCR. We do not know why the validation rate was this low. Although we used the same strains and similar conditions, it is likely that subtle variations in the worm populations or their environment, their synchronization, or the heat-shock protocol led to changes in experimental conditions. We have not seen similar large scale qPCR validations for other *C. elegans* microarray experiments, so we cannot comment on whether this is a common phenomenon; however two other microarray analyses performed in our lab utilizing heat-shock expression of a transcription factor yielded similar validation rates (<50%; J. Siegel and D. Eisenmann; L. Gorrepati, and D. Eisenmann, unpublished results). Of these 39 genes, 22 were regulated as predicted following conditional expression of gain-of-function BAR-1 and dominant negative POP-1 proteins. Four of these 22 genes appeared to be targets of a DAF-16/BAR-1 complex, since they were not up-regulated after expression of ΔNTBAR-1 in a *daf-16(mgDf50)* background and were not considered further. Finally, of the remaining genes, 14 showed decreased expression in animals carrying a loss-of-function mutation in *bar-1/*beta-catenin gene, and 11 of these showed increased expression in animals carrying a mutation in the *pry-1* gene, which encodes the Wnt pathway negative regulator Axin. Since these genes show regulation by Wnt signaling in both gain-of-function (*delNTBAR-1*, *delNTPOP-1*) and loss-of-function [*bar-1(ga80)*, *pry-1(mu38)*] Wnt mutant backgrounds, we consider these 11 genes, *bli-1*, *col-38*, *col-49*, *col-71*, *dao-4*, *F08G2.7*, *pry-1*, *sptf-2*, *tag-164(ok771)*, *Y41C4A.11*, and *Y43C5A.3* likely to be direct targets of the WBC pathway in *C. elegans*.

We examined the normal temporal and spatial expression of this subset of genes and found that a small group of them (*bli-1*, *col-38*, *col-49*, *col-71*, *dao*-4, and *Y41C4A.11)* showed a peak of expression in the mid L4 stage. We created transcriptional reporters for these genes, and as expected for collagen-encoding genes, we observed expression of *bli-1*, *col-38*, *col-49*, and *col*-71 reporters in hypodermal cells. The unknown gene *dao-4* showed a similar expression pattern. The L4 stage peak expression of *bli-1*, *col-38*, *col-49*, and *col-71* in hypodermal cells suggests these genes are likely to be used in synthesis of the adult cuticle. Other Wnt responsive genes with similar temporal and spatial regulation such as *dao-4* and *Y41C4A.11*, may also be involved in this process. We examined this small group of genes for phenotypes suggestive of a role in cuticle synthesis or function and found several phenotypes in RNAi-treated larvae that are consistent with that function, such as Bli, Dpy, Mlt, and defects in cuticle permeability. Significantly, *bar-1(ga80)* mutant animals also showed a defect in the cuticle permeability assay, indicating that reduction of function for this Wnt pathway component caused the same phenotype as reduction of function for putative BAR-1 targets. Finally, both expression of the endogenous genes and the penetrance of expression of transcriptional reporter constructs for three of these collagen genes were sensitive to alterations in Wnt pathway signaling in the L3 and L4 stages. The fact that a group of L4-expressed collagen genes are responsive to Wnt signaling suggests a previously unknown function for Wnt signaling in the regulation of adult cuticle synthesis.

Several major questions remain unresolved from the preliminary analysis here. First, although these genes increase in expression within an hour of ectopic activation of Wnt signaling, and show decreased expression in *bar-1* mutants, we have not yet been able to show that these genes are direct targets of the Wnt pathway. The upstream regions for the 22 genes w characterized in detail (Table 1) all have two or more predicted POP-1/TCF binding sites; in fact 20 of 22 have more observed POP-1 binding sites than predicted based on the GC content of the *C. elegans* genome (Table S9). Unfortunately, chromatin immunoprecipitation experiments using existing POP-1 reagents have not proven to be reliable in our hands. Future experiments addressing the binding of POP-1 *in vitro* and *in vivo* to sites in the promoters of these genes should help address whether these genes are direct targets of POP-1/BAR-1. Second, using modENCODE project RNASeq data across developmental stages, we identified 40 *col* genes showing a peak of expression in the L4 stage; therefore, the four Wnt-responsive col genes we identified are only fraction of the collagen genes expressed strongly at this time. It is possible that additional *col* genes others are also regulated by Wnt signaling but did not meet our criteria. Still, it is not clear why only a subset of *col* genes expressed for use in the adult cuticle would be regulated by Wnt signaling. Third, although these genes are regulated by Wnt signaling in the L4 stage, it is also clear that Wnt signaling is not solely responsible for their expression, since their L4 expression does not disappear in a *bar-1(ga80*) mutant background, but is only reduced. Additional factors are likely to act with the Wnt pathway to control the spatial [hypodermal cell type; see (Gilleard *et al.* 1997)] and temporal [mid L4 stage; see (Liu *et al.* 1995; Rougvie and Ambros 1995)] expression of these col genes in the hypodermal cell types at the L4 stage. Fourth, although we hypothesize that Wnt signaling plays a role in the regulation of expression of these genes at the L4 stage, the source and identity of the Wnt signaling tissue or cells that these genes are responding to is currently unknown. Finally, the similar spatial and temporal expression pattern of the gene *dao-4*, as well as the low penetrance Dpy phenotype caused by *dao-4* RNAi suggests this gene may function in the process of adult cuticle synthesis, but the function of the novel protein encoded by *dao-4* is currently unknown.

Our goal here was to extend our knowledge of Wnt signaling in *C. elegans* by identifying genes regulated by the WBC or “canonical” Wnt signaling pathway in that organism during larval development. We used conditional expression of a gain-of-function BAR-1 beta-catenin protein to activate Wnt signaling at the L2/L3 molt and found 104 genes that showed increased expression, and we went on to identified a small number of the Wnt-regulated genes, including a cluster of collagen-encoding genes, that are highly expressed in the fourth larval stage during normal development. Therefore, even in light of the aforementioned unanswered questions, we believe the use of a gain-of-function beta-catenin reagent to ectopically activate Wnt signaling was successful at identifying additional Wnt-regulated target genes. These genes are likely to represent *bona fide* WBC pathway target genes, and therefore these results extend the small list of known Wnt targets in *C. elegans*.

## Supplementary Material

Supporting Information
