# Supplementary material for: Use of an Activated Beta-Catenin to Identify Wnt Pathway Target Genes in Caenorhabditis elegans, Including a Subset of Collagen Genes Expressed in Late Larval Development
Source: G3 (Bethesda). 2014 Feb 25;4(4):733–47. doi: 10.1534/g3.113.009522 (PMC4059243; doi:10.1534/g3.113.009522)
Supplement: Supporting Information [file supp_4_4_733__index.html]

Use of an Activated Beta-Catenin to Identify Wnt Pathway Target Genes in Caenorhabditis elegans, Including a Subset of Collagen Genes Expressed in Late Larval Development — Supporting Information 

# Use of an Activated Beta-Catenin to Identify Wnt Pathway Target Genes in *Caenorhabditis elegans*, Including a Subset of Collagen Genes Expressed in Late Larval Development

## Supporting Information for Jackson *et al.*, 2014

**Files in this Data Supplement:**

- Supporting Information - Figures S1-S2 and descriptions of Files S1-S2 and Tables S1-S9 (PDF, 1 MB)
- Figure S1 - YFP reporter expression for *pry-1, Y41C4A.11* and *sptf-2*. (PDF, 457 KB)
- Figure S2 - Cuticle integrity assay. (PDF, 734 KB)
- File S1 - *col* genes expression data (.zip, 193 KB)
- File S2 - L4 peak genes data (.zip, 101 KB)
- Table S1 - BAR-1 responsive genes identified by microarray (.zip, 60 KB)
- Table S2 - BAR-1 responsive genes in clusters (.zip, 30 KB)
- Table S3 - GO term statistics (.zip, 34 KB)
- Table S4 - Microarray data for known Wnt targets (.zip, 32 KB)
- Table S5 - YFP transcriptional reporter constructs (.zip, 35 KB)
- Table S6 - Stage-specific col genes (.zip, 47 KB)
- Table S7 - L4-peak gene identities (.zip, 55 KB)
- Table S8 - Oligonucleotides used (.zip, 32 KB)
- Table S9 - POP-1 binding sites in targets (.zip, 15 KB)
