# Supplementary material for: Use of an Activated Beta-Catenin to Identify Wnt Pathway Target Genes in Caenorhabditis elegans, Including a Subset of Collagen Genes Expressed in Late Larval Development
Source: G3 (Bethesda). 2014 Feb 25;4(4):733–47. doi: 10.1534/g3.113.009522 (PMC4059243; doi:10.1534/g3.113.009522)
Supplement: Supporting Information [file supp_g3.113.009522_009522SI.pdf]

G3 · Genes | Genomes | Genetics

# Use of an activated beta-catenin to identify Wnt pathway target genes in *Caenorhabditis elegans*, including a subset of collagen genes expressed in late larval development

**Belinda M. Jackson[*, 1], Patricia Abete-Luzi[*], Michael W. Krause[#] and David M. Eisenmann[*]**

[*] Department of Biological Sciences, University of Maryland Baltimore County, Baltimore, MD USA 21250
[#] Laboratory of Molecular Biology, NIDDK, National Institutes of Health, Bethesda, MD 20892-0510
[1] present address: Department of Medicine, Uniformed Services University of the Health Sciences, Bethesda, MD 20814

dataset: GSE51502 (GEO)

corresponding author: David M. Eisenmann
Department of Biological Sciences
University of Maryland, Baltimore County
1000 Hilltop Circle
Baltimore, MD 21250
(410)455-2256
eisenman@umbc.edu

**DOI: 10.1534/g3.113.009522**

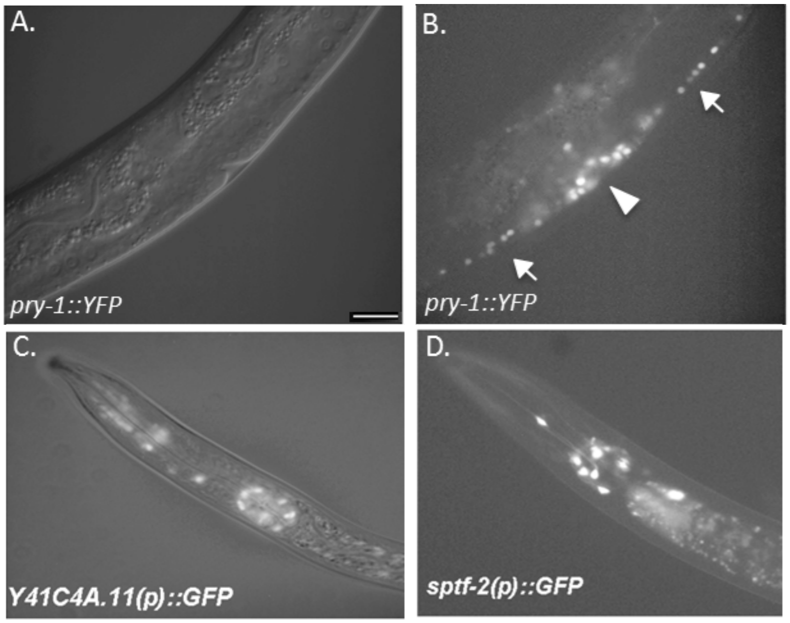


**Figure S1 YFP reporter expression for *pry-1, Y41C4A.11 and sptf-2.*** (A) and (B) expression of *pry-1::YFP* transcriptional reporter in midbody region of an L4 hermaphrodite showing expression in vulval cells (arrowhead) and ventral neurons (arrows). (A) Nomarski image; (B) fluorescence image. (C) and (D) merged Nomarski and fluorescence images of the head region of L4 stage hermaphrodites showing expression from transcriptional reporters for (C) *Y41C4A.11* and (D) *sptf-2.*

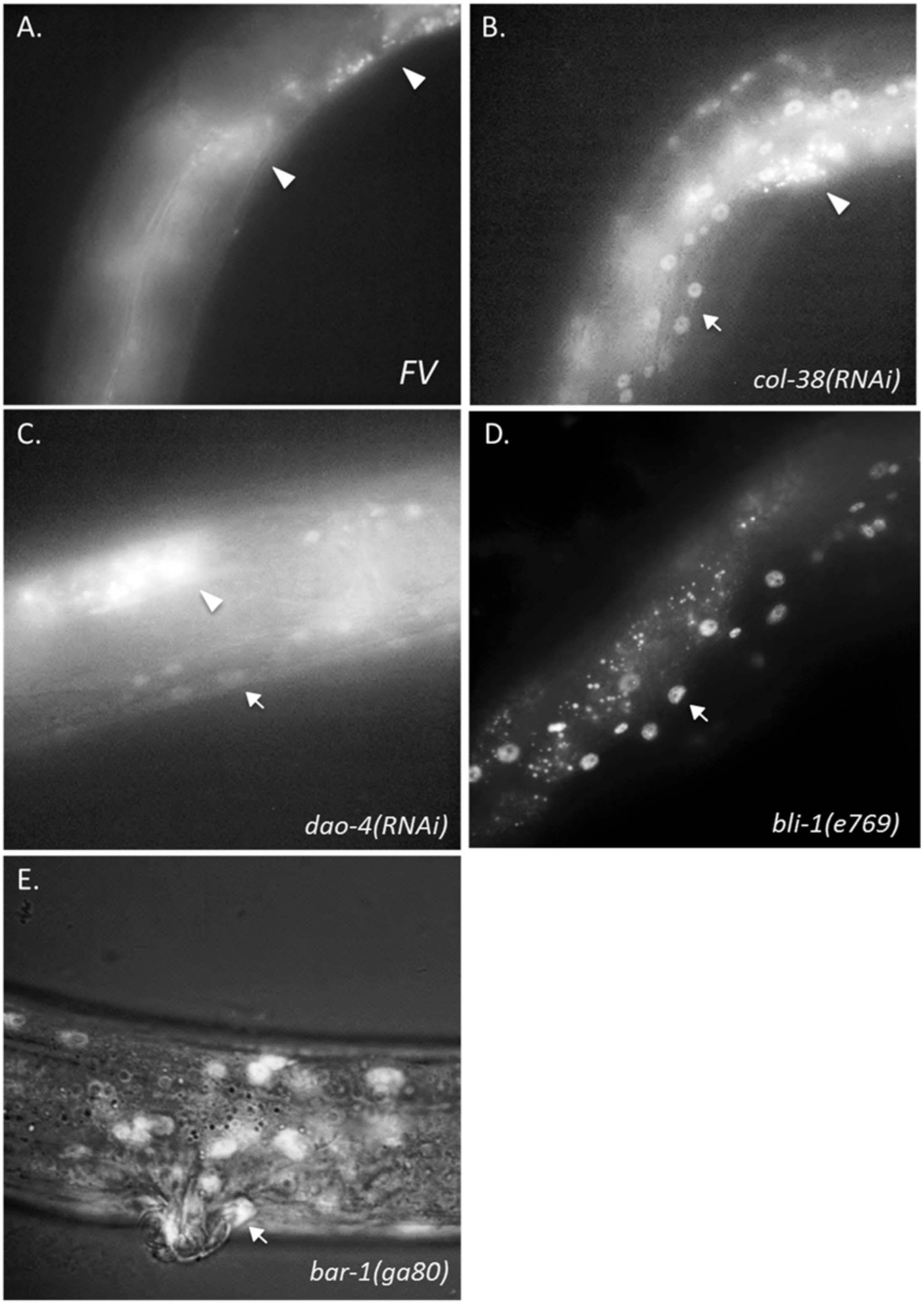


**Figure S2 Cuticle integrity assay.** Cuticle integrity was assayed in adult animals by permeability to Hoechst stain (Moribe *et al.* 2004). (A) feeding vector (FV) control animal shows only background staining and intestinal autofluorescence (arrowheads). (C - E) *col-38(RNAi), dao-4(RNAi), bli-1(e769)* and *bar-1(ga80)* animals show nuclear staining (arrows) throughout the body not seen in control animals.

**File S1**

***col* genes expression data**

Available for download at http://www.g3journal.org/lookup/suppl/doi:10.1534/g3.113.009522/-/DC1

**Worksheet 1 'all cols'.** A set of 188 *C. elegans* 'collagen' genes (*col* genes; although not all gene names begin *col*) was identified as the union of those genes with either 'nematode cuticle collagen' (IPR002486; 164 genes) or 'collagen triple helix' (IPR008160; 160 genes) as an InterPro domain (http://www.ebi.ac.uk/interpro/). (A) Wormbase gene ID number, (B) molecular/genetic gene names, (C) physical sequence names (column C).

**Worksheet 2 'col developmental data'.** modENCODE RNASeq gene expression data from ((Gerstein *et al.* 2010))was examined for the total expression of 184 *col* genes at seven developmental timepoints. (A) molecular/genetic gene names; (B) physical sequence names; (C-I) raw data for expression of gene at early embryo, late embryo, L1, L2, L3, L4, Adult; (J) absolute value for the stage with highest expression; (K) total of data from late embryo to young adult; (L-Q) percent of the total expression observed in each stage, late embryo to adult; (S) value for stage with the highest percent of the total expression; (T-V) values for next three highest stages. Data were sorted by 'highest percent total expression' (S) to identify those *col* genes for which 50% or more of the total developmental expression was observed at one time point (114/184). The stage of peak developmental expression ($\geq$ 50% of total expression) is indicated (X). Some genes lack expression data for the early embryo, late embryo and L1.

**Worksheet 2 'l emb'.** *col* genes showing at least 50% of total developmental expression in the late embryo stage. Columns are as indicated above.

**Worksheet 3 'L1'.** *col* genes showing at least 50% of total developmental expression in the L1 stage. Columns are as indicated above.

**Worksheet 4 'L2'.** *col* genes showing at least 50% of total developmental expression in the L2 stage. Columns are as indicated above.

**Worksheet 5 'L3'.** *col* genes showing at least 50% of total developmental expression in the L3 stage. Columns are as indicated above.

**Worksheet 6 'L4'.** *col* genes showing at least 50% of total developmental expression in the L4 stage. Columns are as indicated above.

**Worksheet 7 'YA'.** *col* genes showing at least 50% of total developmental expression in the young adultstage. Columns are as indicated above.

**Worksheet 8 'dauer'.** *col* genes showing a level of gene expression in the dauer larval at least three fold greater than the highest level of gene expression during any stage in development. Columns (A – J) are as indicated above. To identify *col* genes expressed in the dauer larva, we used data from *daf-2* mutant animals entering and exiting dauer from ((Gerstein *et al.* 2010))(columns K – M). Those genes for which the absolute value of expression in the 'entering dauer' animals (column L) was at least three fold higher than the highest level of expression seen at any stage of development (embryo –> Adult) are indicated, with the ratio of 'dauer level'/'highest developmental level' shown (column O).

**Worksheet 9 'by total'.** The *col* gene developmental data sorted by the total level of expression across all developmental timepoints (column K).

**File S2**

**L4 peak genes data**

Available for download at http://www.g3journal.org/lookup/suppl/doi:10.1534/g3.113.009522/-/DC1

modENCODE RNASeq gene expression data from ((Gerstein *et al.* 2010)) was examined for the total expression of all genes at seven developmental timepoints. (A) physical sequence names; (B-H) raw data for expression of gene at early embryo, late embryo, L1, L2, L3, L4, Adult; (I) total of data from late embryo to young adult; (K) Percentage of total gene expression seen in L4 was calculated, and those genes showing greater than 50% of their total developmental expression in the L4 stage are shown.

**Tables S1-S9**

**Available for download at http://www.g3journal.org/lookup/suppl/doi:10.1534/g3.113.009522/-/DC1**

**Table S1 BAR-1 responsive genes identified by microarray.** Genes showing a statistically-significant change in gene expression of two fold or greater in response to expression of delNTBAR-1 protein. 104 upregulated genes (top) and 62 downregulated genes (bottom) are listed. Columns: (A) physical sequence name, (B) genetic or molecular name (if any), (C) known protein homology, protein domains, genetic or molecular data, (D) average fold change (triplicate) in expression between *HS::delNTbar-1* and *hs:control* strains, (F) p value. In the first worksheet, genes are sorted by fold change. In the second worksheet, genes are sorted into broad categories of function. The third worksheet gives Wormbase gene IDs for each gene.

**Table S2 BAR-1 responsive genes in clusters.** BAR- responsive genes that are located on the same cosmid. Upregulated genes above, downregulated genes below. Genes in bold are adjacent (no intervening genes). Columns: (A) physical sequence name, (B) genetic or molecular name (if any), (C) known protein homology, protein domains, genetic or molecular data, (D) average fold change (triplicate) in expression between *HS::delNTbar-1* and *hs:control* strains, (E) p value. * indicates possible crosshybridization of probe sets between these genes.

**Table S3 GO term statistics.** Identification of overrepresented and underrepresented GO functional terms for BAR- responsive genes (uppregulated genes above, downregulated genes below). Analysis was carried out using FuncAssociate at http://llama.mshri.on.ca/cgi/func1/funcassociate (Berriz, et al., 2009). N = number of genes with attribute in dataset; X = total number of genes with attribute; LOD = logarithm of odds; P = p value; P adj = resampling adjusted p value; attrib ID = Gene Ontology attribute; attrib name = Gene Ontology attribute name.

**Table S4 Microarray data for known Wnt targets.** Columns: (A) gene name, (B) Wnt pathway that regulates the gene (WBC = Wnt/BAR-1 canonical pathway; WBA = Wnt/beta=catenin asymmetry pathway), (C) time of development when regulation is known to occur, (D) reference, (E) average fold change (triplicate) in expression between *HS::delNTbar-1* and *hs:control* strains, (F) p value, (G) number of triplicates in which a statistically significant Increase or Decrease call was made.

**Table S5 YFP transcriptional reporter constructs.** Column A) gene; column B) amount of upstream DNA (relative to gene ATG) used to make transcriptional fusion to YFP coding sequences; column C) for *col-71* and *Y41C4A.11* additional sequences were needed to see YFP expression. In both cases, the genomic DNA from the ATG to the start of exon 3 (therefore including exons 1 and 2, introns 1 and 2) was also included in the fusion.

**Table S6 Stage-specific col genes.** modENCODE RNASeq gene expression data was analyzed for the expression of 184 *C. elegans* genes with 'collagen triple helix repeat' (IPR008160) or 'nematode cuticle collagen' (IPR002486) as a protein domain. The total expression for six developmental stages (late embryo, L1, L2, L3, L4, Adult) was summed for each gene, and the percent of the total expression observed in each stage was calculated (see Supplemental Data file 1). Genes showing greater than 50% of their total developmental expression in any stage (128/185 genes) are indicated in the column for each stage (number of genes is indicated in parenthesis). The column to the right indicates the percentage total expression observed in that stage. To identify *col* genes expressed in the dauer larva, we used data from *daf-2* mutant animals entering and exiting

dauer. Those genes for which expression in the 'entering dauer' animals was three fold or more higher than the highest level of expression at any stage of development (embryo –> Adult) are indicated, in the last column. These data are indicated graphically in Figure S2. Genes shown in bold were identified as 'BAR-1 responsive genes' (Table S1).

**Table S7 L4-peak gene identities.** Columns A and B: 703 genes from the modENCODE data set (GERSTEIN *et al.* 2010) that show 50% or more of their expression in the L4 stage were identified (File S2), and are indicated by physical sequence name (A) and molecular or genetic gene name (B). Columns D and E: 72 genes that show 75% or more of their expression in the L4 stage, indicated by physical sequence name (D) and molecular or genetic gene name (E). Genes indicated in blue are BAR-1 responsive genes identified by microarray analysis (Table S1).

**Table S8 Oligonucleotides used**

**Worksheet 1.** Oligonucleotides used for qPCR validation of candidate Wnt target genes.

**Worksheet 2**. Oligonucleotides used to create YFP reporters by Gateway cloning or overlap extension. Oligonucleotides used to amplify a portion of the *bli-1* gene for RNAi by feeding.

**Table S9 POP-1 binding sites in targets.** We examined the upstream regions of 38 validated BAR-1 targets (Columns A and B) for the presence of POP-1 binding sites. The 22 genes from Table 1 are indicated in red. Column C indicates the observed fold upregulation of each gene in the microarray analysis. Column D indicates the length of upstream region analyzed (from gene ATG to the transcript of next gene upstream). Column E indicates the number of predicted POP-1 binding sites based on a sequence of that length, the GC content of the *C. elegans* genome, and a POP-1 consensus site (YTTTGWWV) based on previously known POP-1 binding shown in vivo (see JACKSON AND EISENMANN 2012). This consensus site is predicted to be found once every 1214 bp. Column F shows observed sites matching the consensus POP-1 site. Green cells indicate genes with more than the predicted number of POP-1 sites. Average values for predicted and observed sites are indicated below columns E and F. Note that a few genes show an excessive number of POP-1 binding sites (e g., *Y71D11A.3, pry-1*); this is due to repetitive DNA elements in the upstream region of these genes which were not masked during the search and which contain sequences matching the consensus POP-1 site.
