# Supplementary material for: Use of an Activated Beta-Catenin to Identify Wnt Pathway Target Genes in Caenorhabditis elegans, Including a Subset of Collagen Genes Expressed in Late Larval Development
Source: G3 (Bethesda). 2014 Feb 25;4(4):733–47. doi: 10.1534/g3.113.009522 (PMC4059243; doi:10.1534/g3.113.009522)
Supplement: Supporting Information [file supp_g3.113.009522_FigureS1.pdf]

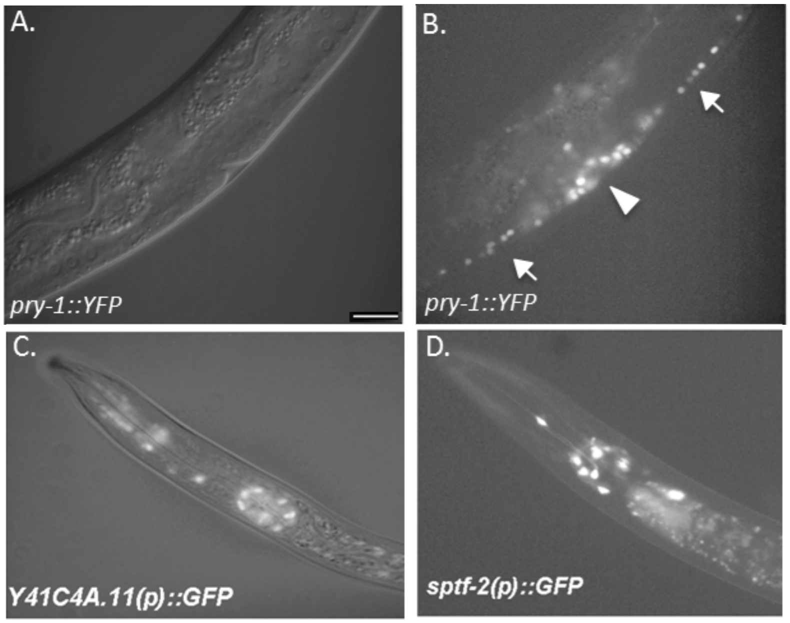

Figure S1 YFP reporter expression for *pry-1, Y41C4A.11 and sptf-2.* (A) and (B) expression of *pry-1::YFP* transcriptional reporter in midbody region of an L4 hermaphrodite showing expression in vulval cells (arrowhead) and ventral neurons (arrows). (A) Nomarski image; (B) fluorescence image. (C) and (D) merged Nomarski and fluorescence images of the head region of L4 stage hermaphrodites showing expression from transcriptional reporters for (C) *Y41C4A.11* and (D) *sptf-2.*
