# Supplementary material for: Use of an Activated Beta-Catenin to Identify Wnt Pathway Target Genes in Caenorhabditis elegans, Including a Subset of Collagen Genes Expressed in Late Larval Development
Source: G3 (Bethesda). 2014 Feb 25;4(4):733–47. doi: 10.1534/g3.113.009522 (PMC4059243; doi:10.1534/g3.113.009522)
Supplement: Supporting Information [file supp_g3.113.009522_FigureS2.pdf]

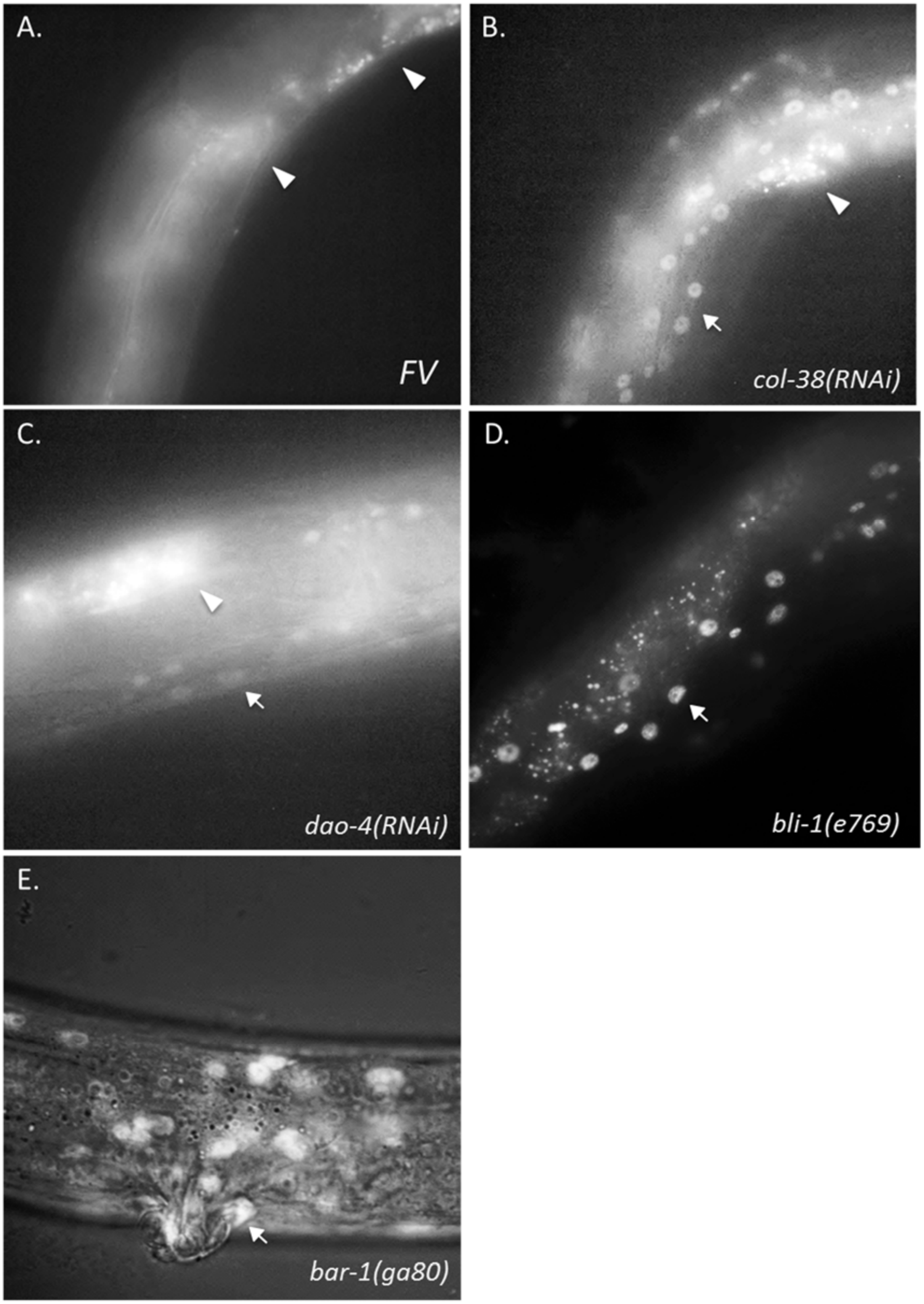

Figure S2 Cuticle integrity assay. Cuticle integrity was assayed in adult animals by permeability to Hoechst stain (MORIBE *et al.* 2004). (A) feeding vector (FV) control animal shows only background staining and intestinal autofluorescence (arrowheads). (C - E) *col-38(RNAi), dao-4(RNAi), bli-1(e769)* and *bar-1(ga80)* animals show nuclear staining (arrows) throughout the body not seen in control animals.
